# Circulating tumor cell and cell-free RNA capture and expression analysis identify platelet-associated genes in metastatic lung cancer

**DOI:** 10.1186/s12885-019-5795-x

**Published:** 2019-06-19

**Authors:** Tim N. Beck, Yanis A. Boumber, Charu Aggarwal, Jianming Pei, Catherine Thrash-Bingham, Patricia Fittipaldi, Ramillya Vlasenkova, Chandra Rao, Hossein Borghaei, Massimo Cristofanilli, Ranee Mehra, Ilya Serebriiskii, R. Katherine Alpaugh

**Affiliations:** 1 Program in Molecular Therapeutics, Fox Chase Cancer Center, Philadelphia, PA 19111 USA; 2 Digestive Disease & Surgery Institute, Cleveland Clinic, Cleveland, OH 44195 USA; 3 Department of Hematology/Oncology, Fox Chase Cancer Center, Philadelphia, PA 19111 USA; 4 Kazan Federal University, Kazan, Russian Federation; 5 Abramson Cancer Center and Division of Hematology/Oncology, Department of Medicine, University of Pennsylvania Perelman School of Medicine, Philadelphia, PA 19104 USA; 6 Genomics Facility, Fox Chase Cancer Center, Philadelphia, PA 19111 USA; 7 Protocol Support Laboratory, Fox Chase Cancer Center, Philadelphia, PA 19111 USA; 8 Janssen Diagnostics LLC, Valley, Huntingdon, PA 19006 USA; 9 Feinberg School of Medicine, Robert H Lurie Comprehensive Cancer Center, Chicago, IL 60611 USA; 10 Head and Neck Medical Oncology, University of Maryland Greenebaum Comprehensive Cancer Center, Baltimore, MD 21201 USA; 11 Biostatistics Facility, Fox Chase Cancer Center, Philadelphia, PA 19111 USA

**Keywords:** NSCLC, SCLC, Circulating tumor cells, Cell-free RNA, Platelets

## Abstract

**Background:**

Circulating tumor cells (CTC) and plasma cell-free RNA (cfRNA) can serve as biomarkers for prognosis and treatment response in lung cancer. One barrier to the selected or routine use of CTCs and plasma cfRNA in precision oncology is the limited quantity of both, and CTCs are only seen in metastatic disease. As capture of CTCs and plasma cfRNA presents an opportunity to monitor and assess malignancies without invasive procedures, we compared two methods for CTC capture and identification, and profiled mRNA from CTCs and plasma cfRNA to identify potential tumor-associated biomarkers.

**Methods:**

Peripheral blood was collected from ten patients with small cell lung cancer (SCLC), ten patients with non-small cell lung cancer (NSCLC) and four healthy volunteers. Two methods were used for CTC capture: the standard epithelial cell adhesion molecule (EpCam) CellSearch kit (unicapture) and EpCAM plus HER2, EGFR and MUC-1 specific combined ferrofluid capture (quadcapture). For the quadcapture, anti-cytokeratin 7 (CK7) was additionally used to assist in CTC identification. NanoString analysis was performed on plasma cfRNA and on mRNA from combined ferrofluid isolated CTCs. Expression data was analyzed using STRING and Reactome.

**Results:**

Unicapture detected CTCs in 40% of NSCLC and 60% of SCLC; whereas, quadcapture/CK7 identified CTCs in 20% of NSCLC and 80% of SCLC. Bioinformatic analysis of NanoString data identified high expression of a platelet factor 4 (PF4)-related group of transcripts.

**Conclusions:**

Quadcapture ferrofluid reagent did not significantly improve CTC capture efficacy. NanoString analysis based on CTC and plasma cfRNA data highlighted an intriguing PF-4-centric network in patients with metastatic lung cancer.

**Electronic supplementary material:**

The online version of this article (10.1186/s12885-019-5795-x) contains supplementary material, which is available to authorized users.

## Background

Lung cancer is the leading cause of cancer-related mortality in both men and women in the United States [1]. Non-small cell lung cancer (NSCLC) accounts for approximately 85% of all lung cancer cases [2]; the majority of patients with NSCLC present with distant metastases, for which chemotherapy continues to be the mainstay of treatment [2]. Stage IV metastatic NSCLC has a historic five-year survival rate of 16% [3]. Small cell lung cancer (SCLC) accounts for about 15% of all lung cancer cases and is an aggressive malignancy with frequent and early metastatic events, with a dismal 5-year survival rate of only 7% [4]. Challenges of treating lung cancer include its heterogeneity [5], tumor evolution throughout treatment [5], therapy resistance [6] and detection at advanced stages. For both SCLC and NSCLC, the current intensive search for reliable biomarkers that can guide treatment decision-making and management is limited by the lack of easily accessible tumor specimens. Nucleic acid secreted by the tumor cells can serve as predictive and prognostic biomarkers [7, 8]. Analysis of circulating tumor cells (CTCs) and circulating cell-free RNA (cfRNA) has shown promise in addressing some of these challenges [8–10] and may provide practical means of surveilling disease status.

CTCs are malignant cells that are found in the peripheral blood once a tumor has become metastatic [11]. These cells may provide surrogate markers to guide decisions regarding prognosis and treatment. CTCs can help in early detection of malignancy and are indicative of metastatic disease, and high levels of CTCs are suggestive of worse outcomes [12]. In more advanced cases, CTCs may provide prognostic information as well as assist in monitoring response to treatment, with utility thus far most notably demonstrated in breast cancer and colorectal cancer [13–16]. In non-small cell lung cancer, CTCs have been shown to be associated with worse progression-free and overall survival [17, 18].

Technological advances have made it possible to use CTCs as a source of tumor DNA/RNA, which can be molecularly profiled to detect informative genomic or transcriptomic signatures and to identify genetic mutations that predict response to targeted therapies [19, 20]. In cases where CTCs are undetectable, an alternative approach is the measurement and analysis of cfRNA in the plasma of patients.

One aim of this study was to compare an alternative combined ferrofluid (quadcapture) capture method to the standard assay (unicapture) and to evaluate the addition of anti-CK7 to enhance the identification of CTCs from patients with NSCLC and SCLC. CTCs can be distinguished from other peripheral blood cells on the basis of their physical and biologic properties [20]. The CellSearch™ Epithelial Cell kit was used for the isolation of CTC by EpCAM specificity (unicapture); it is the gold standard and remains the only FDA-approved test for the capture and identification of CTCs: it utilizes an immunomagnetic separation with epithelial cell adhesion molecule (EpCam) specific ferrofluid [21, 22]. However, some cells may be lost to capture due to low levels or downregulation of EpCAM [23, 24]. We hypothesized that targeting additional tumor specific membrane markers in conjunction with the current EpCAM-ferrofluid (unicapture) CellSearch platform would increase CTC capture and yield. To this end, we assessed a novel immunoferrofluid capture with a novel ferrofluid cocktail of MUC-1, EGFR and HER2 in addition to EpCAM (quadcapture).

A second aim was to define an mRNA expression signature for NSCLC and/or SCLC based on analysis of CTCs and plasma cfRNA using the NanoString digital genomics platform [25], which covers 770 discrete cancer relevant genes. Validation of CTC mRNA and plasma cfRNAs profiles is an underexplored concept and may assist in identifying prognostic and treatment response predictive biomarker signatures and provide the foundation for hypothesis generation and advanced translational research. This effort identified four genes: one involved in immunity (*CCL5*) and three involved in platelet degranulation (*CLU, SPARC* and *SRGN*) as part of a platelet factor 4 (*PF4*)-centric network. The role of platelets in lung tumorigenesis and tumor progression has attracted much interest [26–29] and has been strongly linked to tumor production of PF4 [27].

## Methods

### Patients and study design

**Fig. 1:**
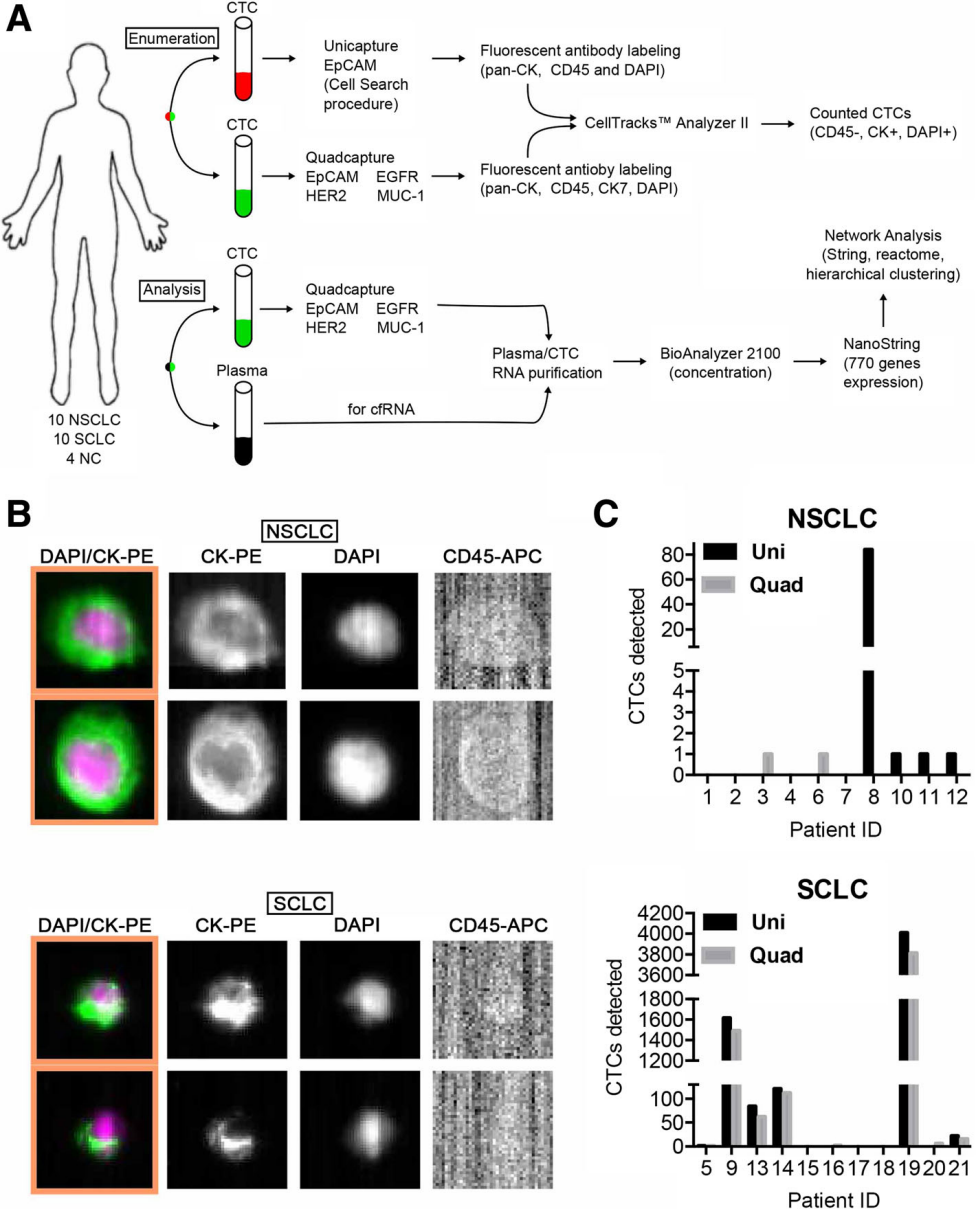
Workflow and CTC/cfRNA capture. **a** Workflow and data analysis. **b** Representative images of captured and fluorescently labeled CTCs from patients with NSCLC (*top*) and SCLC (*bottom*). (**c**) Quantified CTC detection using Uni (unicapture) and Quad (quadcapture) for patients with NSCLC or SCLC. NSCLC = non-small cell lung cancer; SCLC = small cell lung cancer; NC = normal control samples; cfRNA = cell free RNA; CK = cytokeratin (tumor stain); DAPI = nuclear stain; EpCAM = epithelial cell adhesion molecule

The protocol for this study was approved by the Fox Chase Cancer Center (FCCC) Institutional Review Board (IRB), study number 10–043. This prospective study was entirely conducted at FCCC. Twenty patients with either metastatic NSCLC (*n* = 10) or extensive stage SCLC (*n* = 10) and initiating a new therapy, signed consent forms and were enrolled in the protocol. Normal control plasma from four healthy individuals without known chronic conditions or cancer was also collected. Figure 1a summarizes the overall workflow. Nanostring mRNA expression analysis was additionally covered by the FCCC IRB, study number 18–4002.

### CTC capture and processing

To capture CTCs, peripheral blood samples were collected into two 10 mL CellSave Preservative tubes™ (Menarini, Bologna, Italy) and one EDTA tube. All tubes were maintained at ambient temperature until processed – the EDTA tube within 24 h and the CellSave tubes within 96 h of collection. The CellSearch™ Epithelial Cell kits (Menarini, Bologna, Italy) were used for the isolation of CTC by EpCAM specificity (unicapture). In addition, CTCs from one CellSave tube and one EDTA tube were processed using a custom mixture of EpCAM-, HER2-, EGFR- and MUC-1-ferrofluid (quadcapture) prepared by Menarini (Huntington Valley, PA), for this collaborative study. All automated CTC isolations were performed on the CellTracks™ AutoPrep System (Menarini, Bologna, Italy). Data was collected and analyzed on the CellTracks™ Analyzer II (Menarini, Bologna, Italy).

Anti-pan cytokeratin (CKs 8, 18, 19)-PE, anti-CD45-APC and DAPI stain (CellSearch Epithelial Cell kit, Menarini, Bologna, Italy) were used to label CTCs after uni- or quadcapture (Fig. 1a). In addition, an anti-CK7 antibody provided by Menarini (Bologna, Italy) was added to the staining cocktail and used in the CTCs captured using the quadcapture mixture. Immunomagnetic enrichment of CTCs using the CellTracks AutoPrep System has been previously described in detail [30]. Briefly, ferrofluid particles conjugated with anti-EpCAM (unicapture) or EpCAM/MUC-1/EGFR/HER2-captured (quadcaptured) are used to capture CTCs from 7.5 mL of blood via magnetic separation. Captured cells are washed, permeabilized and labeled with fluorescent antibodies. Following labeling, cells are washed, re-suspended in cell fixative and loaded into cartridges. Cartridges are placed in magnetic holders (MagNest, Menarini, Bologna, Italy) which align the ferrofluid-captured cells with the cartridge surface. The MagNests are placed into the CellTracks Analyzer II, where the fluorescently-labeled cells are scanned, and images are captured. Images are sorted by computer-assisted software selecting events based on: negative CD45, positive cytokeratin and positive DAPI. Captured images are displayed as “thumbnails” and reviewed. Images depicting complete cells are selected as a CTC. CTCs for enumeration were defined as EpCAM-captured (unicaptured) or EpCAM/MUC-1/EGFR/HER2-captured (quadcaptured), cytokeratin positive, nuclear stain (DAPI) positive and CD45 negative.

### RNA processing and analysis

The NanoString nCounter PanCancer Progression Panel [25] (NanoString Technologies, Seattle, WA) was used to profile gene expression of 770 genes (Additional file 1: Figure S1 and Additional file 4: Table S1). Briefly, plasma RNA extraction was performed using Norgen plasma/serum RNA purification kit (Cat#55000, Thorold, Canada). Plasma RNA concentration was measured using a BioAnalyzer 2100 (Agilent, Santa Clara, CA). RNA in normal control samples was: mean 44 pg/ul, max 51 and min 41; while in lung cancer specimen it was: mean 400 pg/ul, max 1228 and min 109. Additional Multiplexed Target Enrichment (MTE) was performed before hybridization to the code sets of NanoString’s nCounter PanCancer Progression Panel, in which with the use of SuperScript® VILO, plasma RNA was converted to cDNA, which was then amplified with target-specific primers using TaqMan® PreAmp MasterMix. For the NanoString procedure, capture probes, reporter probes and specimen total RNA were hybridized overnight in a thermocycler, and then were applied to nCounter cartridges. Purification was then processed on the nCounter Prep Station, and finally images were captured on the nCounter Digital Analyzer. Plasma derived specimens used 300 ng of total input RNA.

### cfRNA preparation

Ferrofluid-captured material was placed in 1 mL RNA*later* solution (Qiagen Sciences Maryland USA), and were stored at -80 °C. RNA was prepared for the plasma and ferrofluid samples using a standard kit (http://www.nanostring.com, NanoString Technologies, Seattle, WA) [25]. The single-cell analysis procedure was used for cell free plasma samples to prepare RNA for analysis, which has additional Multiplexed Target Enrichment (MTE) before hybridization, in which with the use of SuperScript® VILO input total RNA was converted to cDNA, and the cDNA was then amplified with target-specific primers using TaqMan® PreAmp MasterMix.

### Statistical methods for CTCs

Allard and colleagues previously found that the rate of patients who had ≥2 CTCs/7.5 ml blood was 20% in a group of patients with NSCLC or SCLC [21], and we used this as the promising rate (i.e. alternative hypothesis). We used 1% as a discouraging rate (i.e. null hypothesis). With a target of 20 patients enrolled in the study, we pre-determined that our novel method would be considered comparable to historical methods if 2 or more of the 20 samples had ≥2 CTCs/7.5 mL of blood. Under this decision rule, our study had 93% power and 1.7% Type I error (one-sided). We used STATA (StataCorp, College Station, Texas) for analyses. Criteria for statistical significance was set to *p*-values < 0.05.

### Analysis of the Nanostring data

Quality control and normalization were performed as recommended by manufacturer (using nSolverAnalysisSoftware version 3.0), and data points with the extreme low counts (<=2) were removed. For each sample, the remaining data points were ranked, to remove the batch effect.

Genes of interest were selected using the 3 biologically relevant scenarios:

A) genes consistently showing higher expression in tumor, than in controls (higher than average in no less than 75% of all tumor samples, while lower than average in no less than 75% of all controls B) rare cancer-specific events were defined as a subset of genes highly ranked (top 10) in at least one tumor sample, but in none of the controls C) a second tier of rare cancer-specific events was defined as a subset of genes which were highly or moderately expressed (top 20 by rank) in at least five tumor samples, but in no more than 1 control sample.

For the subsets of plasma- and CTC-derived nanostring data (using selection criteria outlined above), hierarchical clustering was used to identify top candidates compared to normal control samples. Hierarchical clustering (similarity metric: Euclidean distance, clustering method: complete linkage) generation of the heatmaps was performed using gplots package (Gregory R. Warnes, Ben Bolker, Lodewijk Bonebakker, Robert Gentleman, Wolfgang Huber Andy Liaw, Thomas Lumley, Martin Maechler, Arni Magnusson, Steffen Moeller, Marc Schwartz and Bill Venables (2016). gplots: Various R Programming Tools for Plotting Data. R package version 3.0.1.(https://CRAN.R-project.org/package=gplots).

### Transcriptomic database analysis

The Kaplan-Meier plotter (http://kmplot.com/analysis/index.php?p=service&cancer=lung) was used to access publicly available databases (Cancer Biomedical Informatics Grid (caBIG, https://biospecimens.cancer.gov/relatedinitiatives/overview/caBig.asp)), the Gene Expression Omnibus (GEO, http://www.ncbi.nlm.nih.gov/geo/) and The Cancer Genome Atlas (TCGA, http://cancergenome.nih.gov) from patients with lung cancer. Transcriptomic data from 1926 patient tumors were analyzed in terms of overall survival for genes of interest. Default settings were used and ‘Compute median survival’ was selected. Kaplan-Meier plots were generated and reported median survivals and *p*-values were calculated by Kaplan-Meier Plotter [31].

### STRING analysis of plasma and CTC genes

To assess the potential functional interrelationships within the identified gene set, we conducted STRING [32, 33] analyses to better understand interactions between gene products identified in patient plasma and CTCs (Table 1). Within a subset of eight genes for which the interaction was detected using the default settings, we have narrowed down on the top genes identified as highly expressed in patient samples (*CCL5, CLU, SRGN* and *SPARC*). The expanded 4-gene network was based on the input of these genes, with the basic settings as follows: ‘meaning of network edges: *confidence*’; ‘active interaction source: *experiments, databases, co-expression’*; ‘minimum required interaction score: *high confidence (0.700)*’; ‘max number of interactors to show: *1*^*st*^ *shell – no more than 10 interactors, 2*^*nd*^ *shell – none.*’ For the enhanced visualization of the biologically most relevant groups in the interaction networks, we have used ‘clusters’ setting, ‘kmeans clustering’ was selected with the number of clusters set at two. To test the confidence of interactions between top gene products and PF4, a focused, high confidence (setting at 0.900) network was generated: the input proteins also included PF4; minimum required interaction score was changed to highest confidence (0.900); the maximum number of interactors was kept at none for the 1st shell; and the kmeans clustering was selected with the number of clusters set at one.

**Table 1:** Top transcripts in patients with metastatic lung cancer based on hierarchical clustering

Genes	Plasma detection	CTC detection
**CCL5**	**A,B,C**	**B**
**CLU**	**A,B,C**	**B**
GPX1	**A,B,C**	**B**
AKT3	**A,B,C**	
ARHGDIB	**A,B,C**	
**SPARC**	**A,B,C**	
**SRGN**	**A,B,C**	
TNXB	**A,B,C**	
**THBS1**	**B,C**	**B**
BAI1	**B,C**	
CLDN1	**B,C**	
**EGF**	**B,C**	
FHL1	**B,C**	
PDK1	**B,C**	
PLEKHO1	**B,C**	
RBL1	**B,C**	
RBX1	**B,C**	
**TGFB1**	**B,C**	
GIMAP4	**B**	**B**
**ITGB3**	**C**	**C**
PECAM1	**C**	**B**
LRG1		**B,C**
PKN1		**B,C**

Only genes satisfying at least two cutoff criteria are shown. A, genes consistently showing higher expression based on plasma and CTC analyses; B and C, first and second tier of rare cancer-specific events. See Material and Methods for details. Bold face, genes forming a tight (confidence score > 0.9) cluster by interaction analysis using String database

### Functional pathway analysis using Reactome

Relevant functional pathways for the gene set of interest (*CCL5, CLU, SPARC, SRGN* and *PF4*) were queried using The Reactome Knowledgebase (https://Reactome.org) [34]. Reactome version 63 (released December 18, 2017), which includes 2179 human pathways, 11,426 interactions, 10,996 proteins, 1764 small molecules and 27,694 literature references was used. The five genes of interest were used as input. Four of five genes were identified as expressed in the ‘platelet alpha granule lumen’. The platelet degranulation pathway was further explored, and a list of genes involved in the pathway as well as a list of genes involved specifically in ‘platelet alpha granule contents’ were downloaded. Pictorial representation of ‘platelet degranulation’ was also downloaded from the Reactome platform [34].

## Results

### Patient characteristics

Between January 2011 and February 2012, 21 patients were enrolled: *n* = 10 with SCLC and *n* = 11 with NSCLC (Additional file 5: Table S2). One NSCLC patient (number 017) declined sample collection and no further testing was performed. The patient was not included in the final analysis. As matched controls for the plasma and CTC RNA analysis, we used samples from four healthy volunteers.

### CTC capture

For each patient, both unicapture and quadcapture methods were applied to peripheral blood specimens (Fig. 1a). CTCs were detected and analyzed for NSCLC samples and SCLC and fluorescent labeling of DAPI, CK, and CD-45 was performed (Fig. 1b). Table 2 summarizes results for CTC capture. CTCs were detected in 6 out of 10 NSCLC samples (60%) and 8 out of 10 SCLC samples (80%) by at least one of the two methods (unicapture or quadcapture).

**Table 2:** Circulating tumor cell analysis in NSCLC and SCLC patients

			Number of CTCs/ mL	
Patient #	Diagnosis	Histology, stage, disease site	Unicapture	Quadcapture
001	NSCLC	Adenocarcinoma, IV; bone	0	0
002	NSCLC	Adenocarcinoma, IV; bone	0	0
003	NSCLC	Adenocarcinoma, IV; brain	0	1
004	NSCLC	Adenocarcinoma, IV; bone	0	0
006	NSCLC	Adenocarcinoma, II; lymph nodes	0	1
007	NSCLC	Squamous, IV; brain metastasis	0	0
008	NSCLC	Adenocarcinoma, IV; lymph node metastasis	84	0
010	NSCLC	Adenocarcinoma, IV; brain, bone, adrenal, subcutaneous tissue	1	0
011	NSCLC	Adenocarcinoma, IV; adrenal	1	0
012	NSCLC	Adenocarcinoma, IV; liver	1	0
005	SCLC	Extensive; liver	1	1
009	SCLC	Extensive; liver, adrenal, bone	1615	1491
013	SCLC	Extensive; lymph nodes	84	62
014	SCLC	Extensive; bone, liver, brain	121	112
015	SCLC	Extensive; bone	0	0
016	SCLC	Limited stage	0	2
018	SCLC	Limited stage	0	0
019	SCLC	Extensive; liver	4007	3810
020	SCLC	Limited stage	0	6
021	SCLC	Extensive	22	16

Detectable CTCs for NSCLC ranged from 1 to 84 in number. For NSCLC, the unicapture method identified CTCs in four patients, with one patient having 84 CTCs/mL and the remaining three having 1 CTC. The quadcapture method identified CTCs in two patients, with one CTC each (Fig. 1c). Overall, we observed a trend with decrease in CTC-positive patients with the new method in NSCLC. Using ≥1 CTC positivity cutoff, 4/10 patients were positive by unicapture, while 2/10 were positive by quadcapture, and when using ≥2 CTC positivity cutoff, 1/10 NSCLC was positive with unicapture and none were positive by quadcapture (Table 2).

Detectable CTCs for SCLC ranged from 1 to 4007 in number. The unicapture method identified CTCs in 6 patients, with a range of 1–4007 CTCs (Fig. 1c). The quadcapture method identified CTCs in 8 patients, with a range of 1–3810. All specimens with CTCs that were identified by unicapture were also identified by quadcapture, and the 6 patients demonstrating CTCs by both methods showed similar numbers by both enrichment/detection procedures (Table 2). Overall, we observed a trend with increase in CTC-positive patients with the new method in SCLC. Using ≥1 CTC positivity cutoff, 6/10 patients were positive by unicapture, while 8/10 were positive by quadcapture, and when using ≥2 CTC positivity cutoff, 5/10 NSCLC was positive with unicapture, while 7/10 were positive by quadcapture (Table 2). Nonetheless, the general difference in number of CTCs for the SCLC patients between the two methods was not statistically significant (*P* = 0.37).

The concordance rate between two methods was 60% with ≥1 CTC positivity cutoff and 85% with ≥2 CTC positivity cutoff.

In summary, no significant improvement was seen with the new quadcapture method compared to standard unicapture method. No association was noted between CTC enumeration and the demographic characteristics of age, gender or smoking history.

### NanoString analysis of RNA from plasma and CTCs

**Fig. 2:**
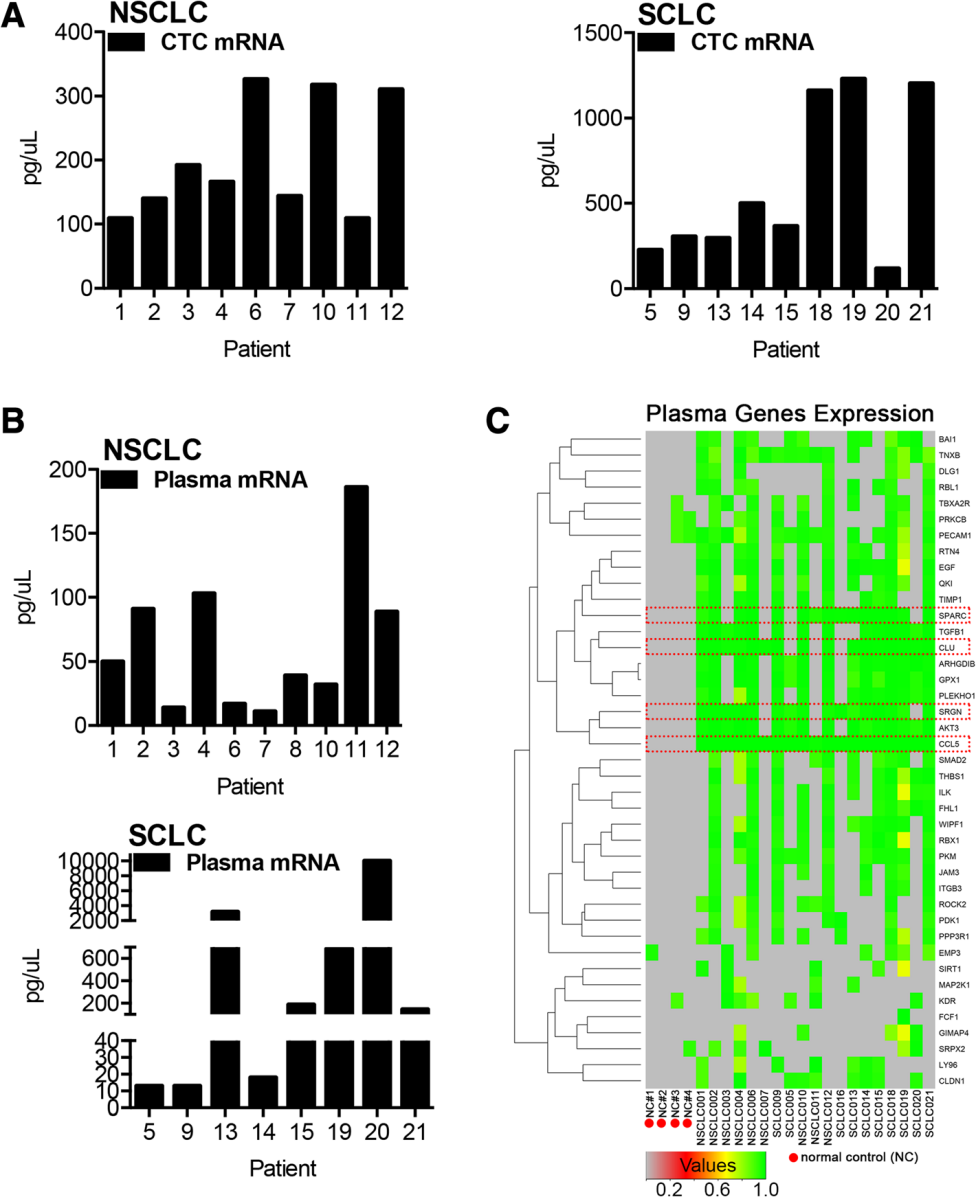
Quantification of detected NSCLC and SCLC mRNA and supervised hierarchical clustering of statistically significant transcripts. **a** Concentration of CTC mRNA for NSCLC (*left*) and SCLC (*right*). **b** Concentration of plasma derived circulating cell free tumor mRNA (cfRNA) from patients with NSCLC (*top*) and SCLC (*bottom*). **c** Heatmap for and hierarchical clustering of 41 transcripts identified as significantly overexpressed in the plasma of patients with NSCLC/SCLC compared to normal control (NC) samples; genes below the detection threshold were set to 0 (gray)

Using samples from the eighteen patients and four normal controls, plasma cfRNA and CTC mRNA was quantified with the single cell PanCancer Progression Panel (Fig. 1). The most significant transcripts are broken down by cfRNA and CTC detection in Table 1 as well as Fig. 2c and Additional file 2: Figure S2.

RNA was quantified from plasma and CTCs for both SCLC and NSCLC samples (Fig. 2a, b). Comparisons of RNA from normal volunteers to RNA from cancer patients identified highly expressed transcripts related to cancer growth, progression and metastasis (Fig. 2c and Additional file 2: Figure S2A).

Expression of a total of 41 genes were statistically significantly and reliably detected in plasma from NSCLC or SCLC patients relative to normal control samples (Fig. 2c and Table 1). The most significantly overexpressed genes based on plasma and CTC mRNA included inflammatory chemokine *CCL5*, and secreted glycoprotein *CLU* (clusterin; Additional file 2: Figure S2A). In addition, two pro-metastatic, pro-invasive, pro-proliferation ligands were represented in the list, including TGFB1 and EGF, the TGF-beta related gene, SRGN and the secreted glycoprotein SPARC (osteonectin) (Fig. 2c and Table 1).

### Focused interaction networks and overall survival

**Fig. 3:**
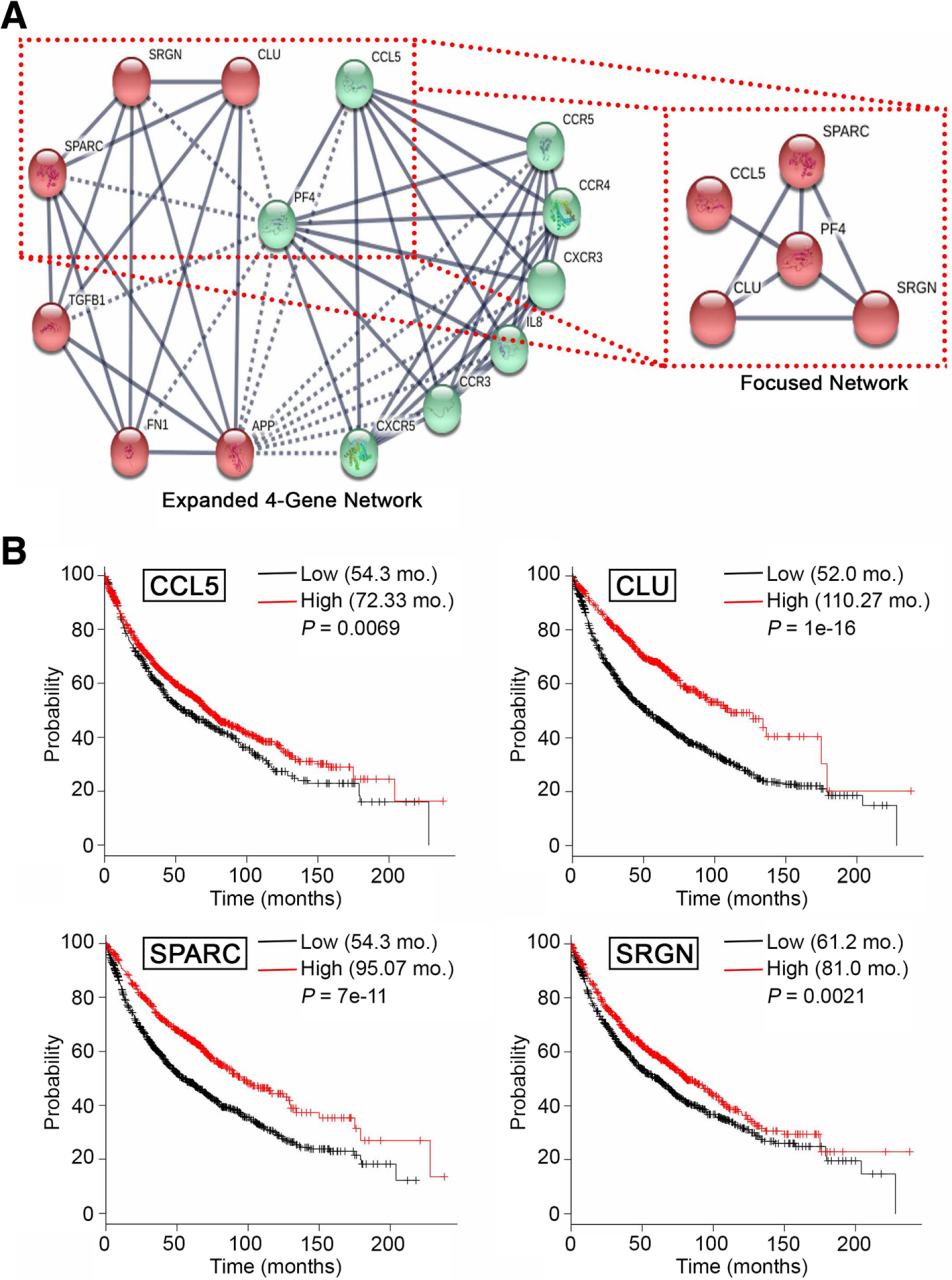
Circulating cancer cell and cfRNA-based interaction network and overall survival. **a** Expanded high confidence (0.700) four-gene (SPARC, SRGN, CLU, CCL5) STRING [32] interaction network (*left*) with the correlating focused network (*right*). **b** Kaplan-Meier overall survival plots based on 1926 transcripts form patients with lung cancer. [31]

Three highly expressed genes were identified with high confidence as present in patient CTCs, and as enriched in patient plasma versus normal control plasma (Table 1): *CCL5, CLU* and *GPX1*. Based on STRING analysis, CCL5 and CLU are components of an interaction network also involving SRGN, SPARC, TGFB1 and several critical inflammatory markers (Fig. 3a). Intriguingly, platelet factor 4 (PF4), an endocrine factor with overexpression correlating with decreased overall survival of patients with lung cancer [27], emerged as a central node connected with high confidence to SRGN, SPARC, CLU and CCL5 (Fig. 3a). PF4 (identified in silico and not part of the 770 gene NanoString platform) and SRGN, SPARC and CLU are functionally associated in the release of platelet alpha granule content (Additional file 3: Figure S3 and Additional file 6: Table S3 and Additional file 7: Table S4).

**Fig. 4:**
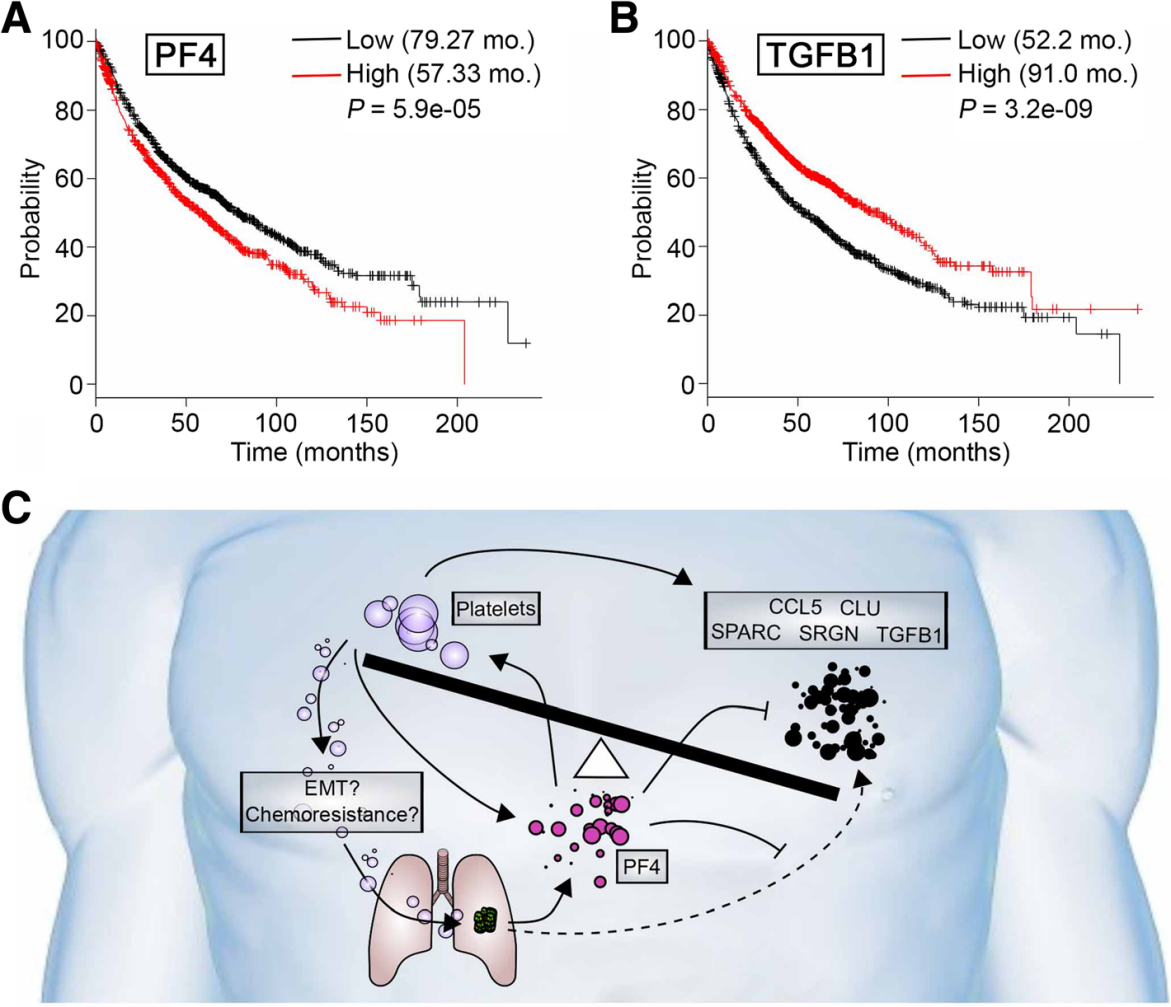
Platelet factor 4 (PF4) survival correlation and proposed model. Kaplan-Meier overall survival for (**a**) PK4 and (**b**) TGFB1 based on transcriptomic data for 1926 lung cancer patient samples. [31] (**c**) Proposed testable model

We next used KM plotter to compare these identified genes to transcriptomic data for 1926 NSCLC specimens [31]. This analysis supported the idea that genes in the PF-4 centered network were overexpressed in patients with poor prognosis (Fig. 3a). For *SRGN, SPARC, CLU* and *CCL5*, higher expression correlated with statistically significant survival differences. Overexpression of each of the four genes detected as highly expressed in patient plasma (Fig. 2c and Table 1) indicated superior survival in a comparison of lung cancer cases (importantly, this is not a comparison of lung cancer to healthy tissue) with high versus low expression (Fig. 3b); specifically, improved survival of 72.33 months versus 54.3 months for cases with high CCL5 (*P* < 0.01); 110.27 months versus 52.0 months for cases with high CLU (*P* < 1E-16); 96.07 months versus 54.3 months for cases with high SPARC (*P* < 7E-11); and 81.0 months versus 61.2 months for cases with high SRGN (*P* < 0.001). Consideration of PF4 in terms of overall survival validated recently reported findings that high expression of PF4 correlates with worse overall survival compared to lower expression [27]. Based on transcriptomic data, overall survival of patients with high PF4 expression was 57.33 months versus 79.27 months for cases with lower expression of PF4 (Fig. 4a). Intriguingly, an additional platelet associated factor, TGFB1 – also identified in patient plasma (Fig. 2c and Table 1) – paralleled CCL5, CLU, SPARC and SRGN survival data, with improved survival seen with TGFB1 expressed at high levels (low expression, 52.2 months; high expression, 91.0 months; *P* = 3.2e-09; Fig. 4b). These findings for the first time, based on CTC and plasma RNA data, propose and intriguing and provocative testable model in metastatic lung cancer, wherein PF4 may act as a negative regulator – likely in terms of platelet activity – of CCL5, CLU, SPARC, SRGN and TGFB1 (Fig. 4c). This intriguing model deserves further study and requires extensive validation.

## Discussion

Precision oncology applies advanced genomic and molecular analyses of tumors to optimize treatment, often relying on target therapy and immunotherapy. An essential component of precision oncology is tracking the response of a tumor to intervention and to adjust treatment accordingly [35]. Precision oncology is only possible with continuous collection and analysis of patient specific data; single genomic biomarkers based on a single – temporal and spatial – tissue biopsy are seldom sufficient to design comprehensive personalized predictive models to accurately guide durable treatment [36]. Intratumoral heterogeneity and tumor evolution [5] are major obstacles of sustained therapeutic responses and possibly curative interventions. Additional factors, including immune-related (e.g., platelets and other immune cells), metabolome-related and microbiome-related, are likely needed to be considered as part of precision oncology. Capture of CTCs and plasma cfRNA presents an opportunity to monitor malignancies without invasive procedures [37]. Leverage of this information through data mining to generate focused interaction networks and to identify potential tumor vulnerabilities and novel treatment angles can help maximize the impact of such data [35]. In this study, we used different approaches to capture and analyze CTCs and RNA from patients with malignant lung cancer and subsequently used advanced data mining to augment our findings and to identify an intriguing PF-4-centric network (Figs. 3a and Fig. 4c).

### CTC capture and CTC-RNA analysis

CellSearch capture reagent (unicapture) is based on EpCAM expression, a proven method of detecting CTCs [9, 38, 39]; however, EpCAM CTC capture has the potential of missing cells that have, for example, undergone EMT [10], a process linked to cancer cell invasion and chemoresistance [6, 40]. A subpopulation of NSCLC and SCLC express, in addition to EpCAM, EGFR, HER2 and/or MUC1; we therefore hypothesized that targeting these additional molecules (quadcapture) would increase the rate of CTC capture.

Overall, quadcapture did not significantly improve the capture of CTCs. For samples that demonstrated CTCs in both assays (uni- and quadcapture), CTC numbers were generally lower in the quadcapture assay. The quadcapture had equal amounts of each ferrofluid: 25% anti-EpCAM, 25% anti-HER2, 25% anti-MUC1 and 25% anti-EGFR. This suggests that EpCAM is potentially essential to capture the maximum number of CTCs, especially for cases with lower than average EpCAM expression. The maximum amount of EpCAM ferrofluid (unicapture) appears capable of capturing CTCs with minimal EpCAM expression. The 75% loss of EpCAM in the quadcapture mixture may have been too steep to realize an additive effect of targeting HER2, MUC1 and EGFR in parallel. Notably, for no patients with NSCLC did both methods identify CTCs (Table 2). The identification of an additional two NSCLC (pts. 003 and 006) and two SCLC (pts. 016 and 020) was potentially enabled by anti-CK7, allowing for the detection of these CTCs in the quadcapture. Other cancers do not have as great CK variance as lung cancer does and the addition of anti-CK7 could enhance lung cancer CTC identification. Due to an absence of accompanying tumor tissue, we were unable to verify dependency on anti-CK7 to detect cytokeratin in these patients. The CellSearch pan cytokeratin reagent does cover the other CKs required for the majority of epithelial cancers. Alternate antibody labeling techniques could be developed in the future by adding biotin-labeled multiple antibodies followed by the maximum amount of streptavidin labelled ferrofluid.

Given that one endpoint of this analysis was to detect ≥2 CTCs in two or more samples with the novel quadcapture ferrofluid, the study did meet its primary endpoint. The presented study is limited by lack of power to compare the number of CTCs detected. More importantly, our study highlights the ability to study RNA expression and novel technologies with small blood samples. As we move further into the era of targeted and immuno-oncology, the ability to study dynamic biomarkers in real time will be increasingly important. Larger studies aimed at CTC subtype classification with distinct molecular features (for instance, EGFR-mutant, KRAS mutant, ALK-rearranged and PD-L1 expressing) are needed.

### Leveraging transcriptomic data and limitations of the gene expression study

Analysis of RNA from plasma and from captured CTCs, using the digital genomics NanoString platform, identified a number of genes that were expressed at higher levels in cancer patients compared to normal controls. These genes may have biologic significance as drivers of metastasis and may indeed be of prognostic relevance.

Advanced data mining and network analysis using STRING [32] and Reactome [34] revealed that four of the identified gene products (for *CCL5, CLU, SPARC* and *SRGN*) interact with PF4, a critical endocrine factor previously described as associated with worse outcome in patients with lung cancer [27]. PF4 functions as a promotor of platelet chemotaxis into the tumor microenvironment and has been linked to carcinogenesis [27]. The roles of platelets in terms of tumor growth, proliferation and metastasis is well-established and provides an important opportunity for possible therapeutic intervention [26–29]. Our analysis proposes a provocative potential model of negative regulation between PF4 and CCL5, CLU, SPARC, SRGN and TGFB1 (Fig. 4c).

Determining the exact source of increased PF4 levels in patient with cancer is complicated. At least three sources for PF4 in have been described. A prominent possibility is the aforementioned overexpression and secretion of PF4 by tumor cell in an endocrine fashion [27]. Alternatively, platelets may express higher levels of PF4 in patients with cancer, which has been described for patients with colorectal cancer; where the level of PF4 in patients with cancer was double that of matched healthy control individuals (Fig. 4c; [41]). Lastly, other myeloid cells, such as dendritic cells or monocytes, could potentially be a source of PF4 [42, 43].

CCL5 and CLU have previously been reported as upregulated and/or secreted in several cancer types. CCL5 is a soluble chemotactic cytokine/chemokine. Interestingly, CCL5 is involved in cancer cell proliferation, metastasis and the formation of an immunosuppressive microenvironment [44]. Circulating CCL5 has also been described as a potential biomarker for tumor load in breast cancer [45]. CLU is a stress-activated, ATP-independent molecular chaperone, normally secreted from cells; it is up-regulated in Alzheimer disease as well as in many tumor types. CLU has also been proposed as a therapeutic target in cancer [46]. Unlike CCL5, CLU is functionally related to genes involved in platelet degranulation (Additional file 3: Figure S3).

Transforming growth factor beta 1 (TGFB1) belongs to the TGF-beta superfamily of cytokines and is a secreted protein with multiple cellular functions, such as regulation of cell growth, proliferation, metastasis and angiogenesis [40, 47]. TGF-beta levels have been shown to correlate with chemotherapy response in NSCLC and have recently been shown to attenuate immunotherapy responses [48]. The TGFB1 pathway is pro-metastatic in late stages of cancer [47, 49, 50] and, like PF4, TGFB1 has been linked to platelet activity in cancer patients [26, 51]. It has also been shown that platelets are a crucial source of bioavailable TGFB1 for tumor cells in the vasculature and for support of tumor cell extravasation [26]. We found TGFB1 RNA significantly elevated in plasma of patients with metastatic lung cancer. Two additional top genes identified in our study were SRGN (serglycine) and SPARC, which mapped to the same functional pathway (i.e., platelet degranulation) that includes TGFB1 and PF4 (Additional file 3: Figure S3) [34].

Survival analyses [31] based on the network of identified genes indicated that increased expression of CLU, CCL5, TGFB1, SRGN and SPARC correlates with improved survival; whereas, high expression of PF4 correlates with reduced survival. A possible model of this apparent paradox is based on the known importance of concentrations of cytokines, a well-described phenomenon for PF4 [52, 53]. For example, at low concentrations, PF4 predominantly occurs as a monomer and acts synergistically with IL-8 in suppressing myeloid progenitor cell proliferation; at sub-stimulatory concentrations PF4 also reduces neutrophil adhesion to endothelium. However, at high concentrations, PF4 forms a tetramer and abrogates IL-8 signaling [52, 53]. Our study leverages data from plasma RNA and CTCs, published studies and focused data mining, to propose a testable model in which high concentration of PF4 induces platelet attraction into the tumor microenvironment and regulates expression and/or availability of CLU, CCL5, TGFB1, SRGN and SPARC (Fig. 4c).

Our proposed model (Fig. 4c) focuses on a proposed central role of PF4. The major sources of PF4 based on established studies are likely from tumor cells and platelets [27, 41–43]. PF4 then promotes downregulation of CCL5, TGFB1, SRGN and SPARC within the tumor microenvironment, immunoregulatory cells, platelets and potentially tumor cells themselves; thus, blunting the established survival benefit higher expression of these genes is associated with [31].

While we detected several potential driver genes which could be the drivers of disease progression and metastasis in lung cancer, several potential limitations of the study should be noted. First, there is the possibility that the some of the mRNAs and associated gene expression signature from plasma and CTCs was in part derived from WBCs. WBCs were contained in the CTC captured product. Plasma from both patients and controls was not double-spun to remove the majority of WBCs. Additional experimental validation of the top genes would be needed using purified WBC from both normal controls and cancer patients to validate the biological function of these genes and clarify their origin. As an example, a recent circulating DNA study demonstrated that a number of mutations identified in the blood of lung cancer patients actually represent clonal hematopoiesis captured from WBCs rather than tumor cell mutations [54]. Second, there is clearly a need for additional independent studies of plasma and CTC mRNAs identification using Nanostring and other platforms to ensure reproducibility of the data. Third, due to inherent limitations of the PCR and capture, reagents with enhanced sensitivity, optimized for blood-based capture need to be developed to improve scientific rigor of these studies, and determination of sensitivity, specificity and validity of these techniques. Additional studies should also be powered to enable comparisons of the number of CTCs detected and eliminated potential technical artifact. However, this study does illustrate the potential of analyzing CTC mRNA as a cornerstone for targeted data mining.

## Conclusion

In summary, we believe that analysis of plasma and CTC mRNA presents a new avenue to advance precision oncology and provides opportunities to generation new hypothesis and translational research. While we identified several possible interactions between PF4 and CLU, CCL5, TGFB1, SRGN and SPARC using STRING [32] and Reactome [34], our model needs careful validation through focused clinical and laboratory-based studies and predominantly serves as an example of leveraging CTC and patient plasma derived data.

## Additional files

Additional file 1: **Figure S1.** Representative molecular categories covered by the 770 gene NanoString platform. (JPG 262 kb)

Additional file 2: **Figure S2.** (*A*) Hierarchical clustering of differentially expressed transcripts based on CTC derived mRNA; differences for 41 genes were statistically significant. (*B*) STRING network of 23 top transcripts. (JPG 311 kb)

Additional file 3: **Figure S3**. Platelet degranulation pathway. Platelet alpha granule contents includes three out of four (CLU, SPARC, SRGN) identified genes as well as PF4 and TGFB1. The figure was generated using Reactome. (JPG 348 kb)

Additional file 4: **Table S1.** List of 770 screened genes. (DOCX 54 kb)

Additional file 5: **Table S2.** Patient and tumor characteristics. (DOCX 17 kb)

Additional file 6: **Table S3.** Complete list of known proteins/compounds involved in platelet degranulation. Data downloaded from Reactome. (DOCX 29 kb)

Additional file 7: **Table S4.** List of known proteins associated with platelet alpha granule content release. Data downloaded from Reactome. (DOCX 22 kb)

## Data Availability

All data is included as part of the manuscript or as part of the supplemental materials section. The datasets used and/or analyzed during the current study are available from the corresponding author on reasonable request.

## Abbreviations

ALK: Anaplastic lymphoma kinase
APC: Adenomatous polyposis coli
ATP: Adenosine triphosphate
CCL5: Chemokine (C-C motif) ligand 5
CD45: Protein tyrosine phosphatase, receptor type, C (also known as PTPRC)
cDNA: Complementary DNA
cfRNA: Cell-free RNA
CK7: Cytokeratin
CLU: Clusterin
CTC: Circulating tumor cells
DAPI: 4′,6-diamidino-2-phenylindole
DNA: Deoxyribonucleic acid
EGF: Epidermal growth factor
EGFR: Epidermal growth factor receptor
EMT: Epithelial-mesenchymal transition
EpCam: Epithelial cell adhesion molecule
FDA: Food and Drug Administration
GEO: Gene Expression Omnibus
GPX1: Glutathione peroxidase 1
HER2: Human epidermal growth factor 2
IL-8: Interleukin 8
KRAS: Kirsten Rat Sarcoma Viral Proto-Oncogene
mRNA: Messenger ribonucleic acid
MTE: Multiplexed Target Enrichment
MUC-1: Mucin 1
NSCLC: non-small cell lung cancer
PD-L1: Programmed death-ligand 1
PF4: Platelet factor 4
RNA: Ribonucleic acid
SCLC: Small cell lung cancer
SPARC: Secreted protein acidic and rich in cysteine
SRGN: Serglycin
TGFB1: Transforming growth factor beta 1
TGF-beta: Transforming growth factor beta
WBC: White blood cell
