# Supplementary figures and images for: Circulating tumor cell and cell-free RNA capture and expression analysis identify platelet-associated genes in metastatic lung cancer
Source: BMC Cancer. 2019 Jun 19;19:603. doi: 10.1186/s12885-019-5795-x (PMC6582501; doi:10.1186/s12885-019-5795-x)

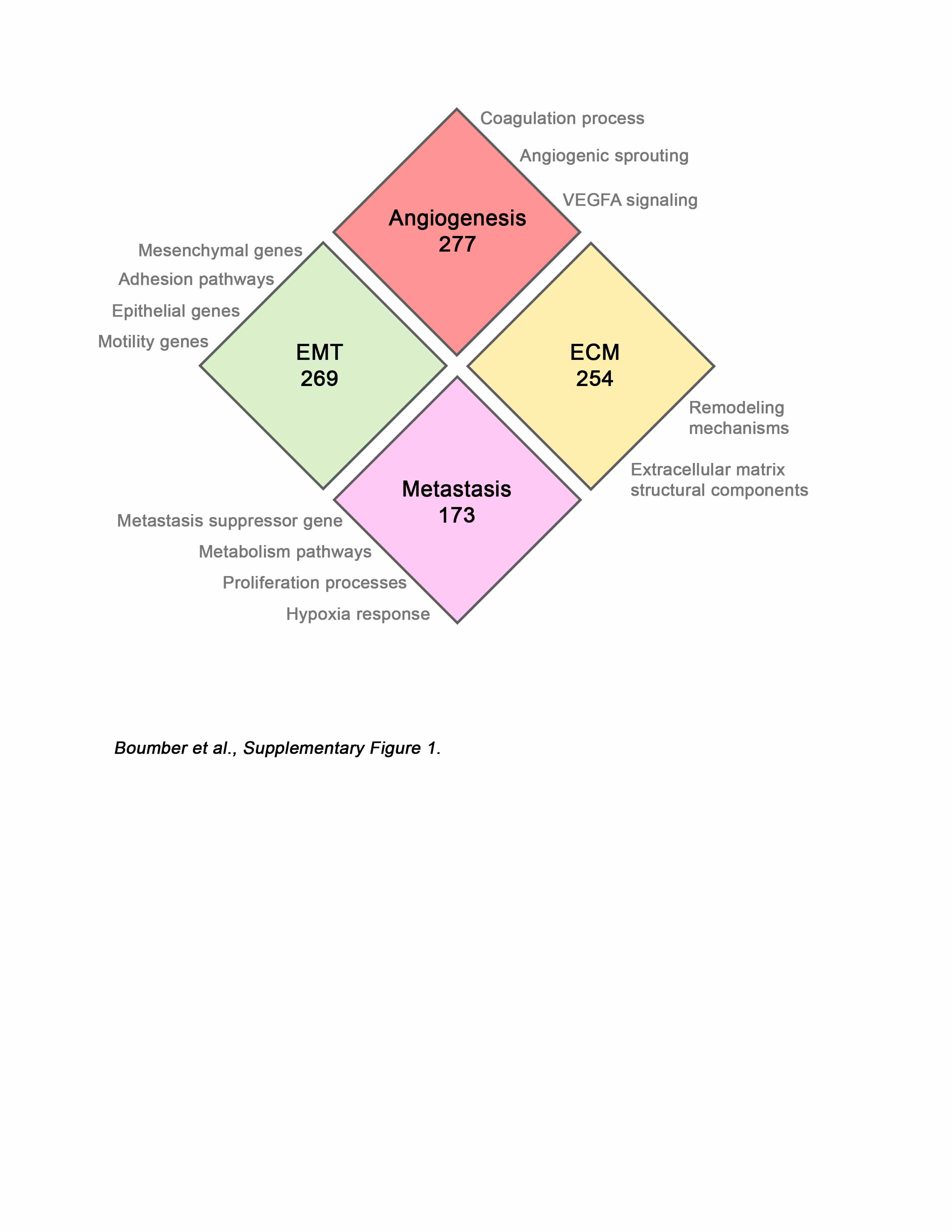

Supplement: Supplementary file 1 — Figure S1. Representative molecular categories covered by the 770 gene NanoString platform. (JPG 262 kb) [file 12885_2019_5795_MOESM1_ESM.jpg]

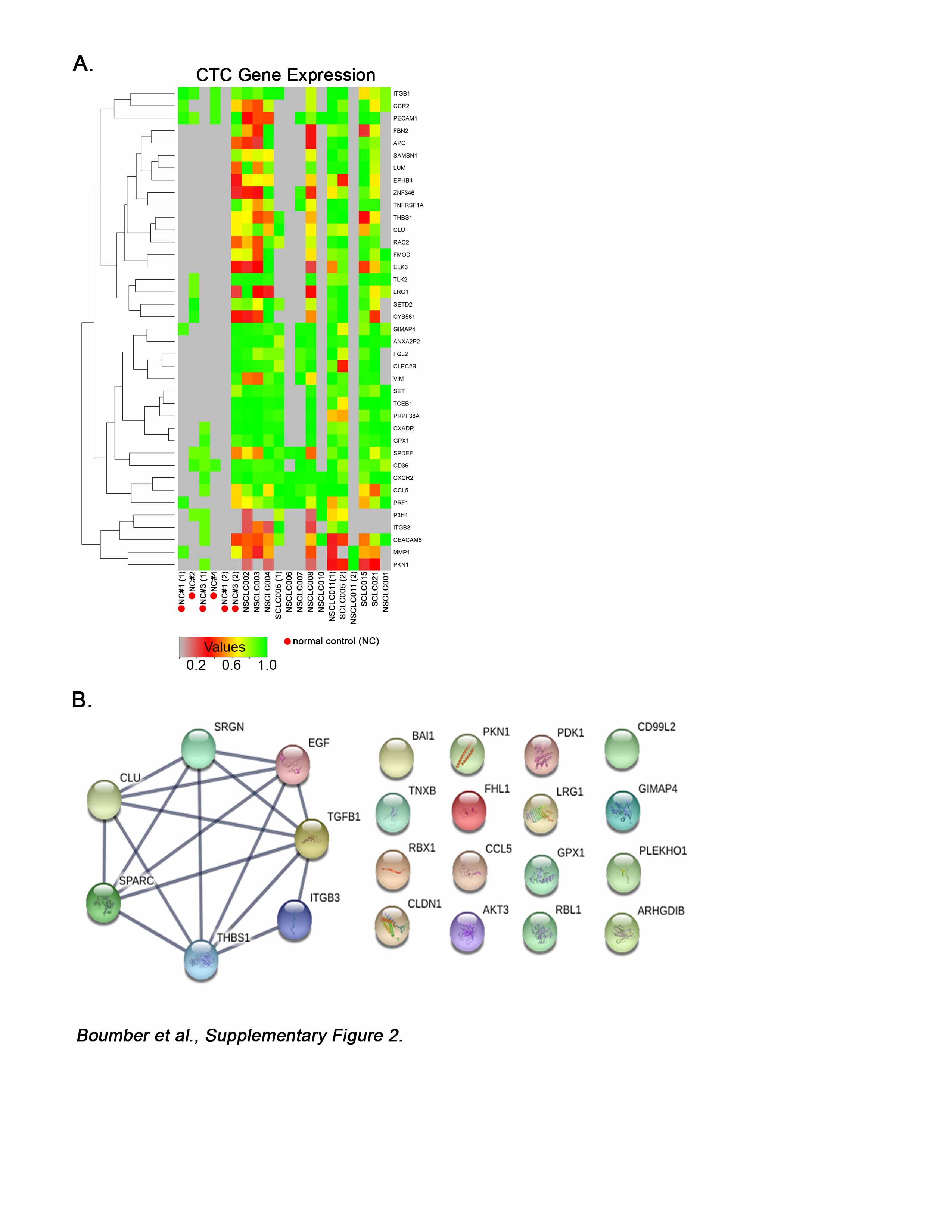

Supplement: Supplementary file 2 — Figure S2. (A) Hierarchical clustering of differentially expressed transcripts based on CTC derived mRNA; differences for 41 genes were statistically significant. (B) STRING network of 23 top transcripts. (JPG 311 kb) [file 12885_2019_5795_MOESM2_ESM.jpg]

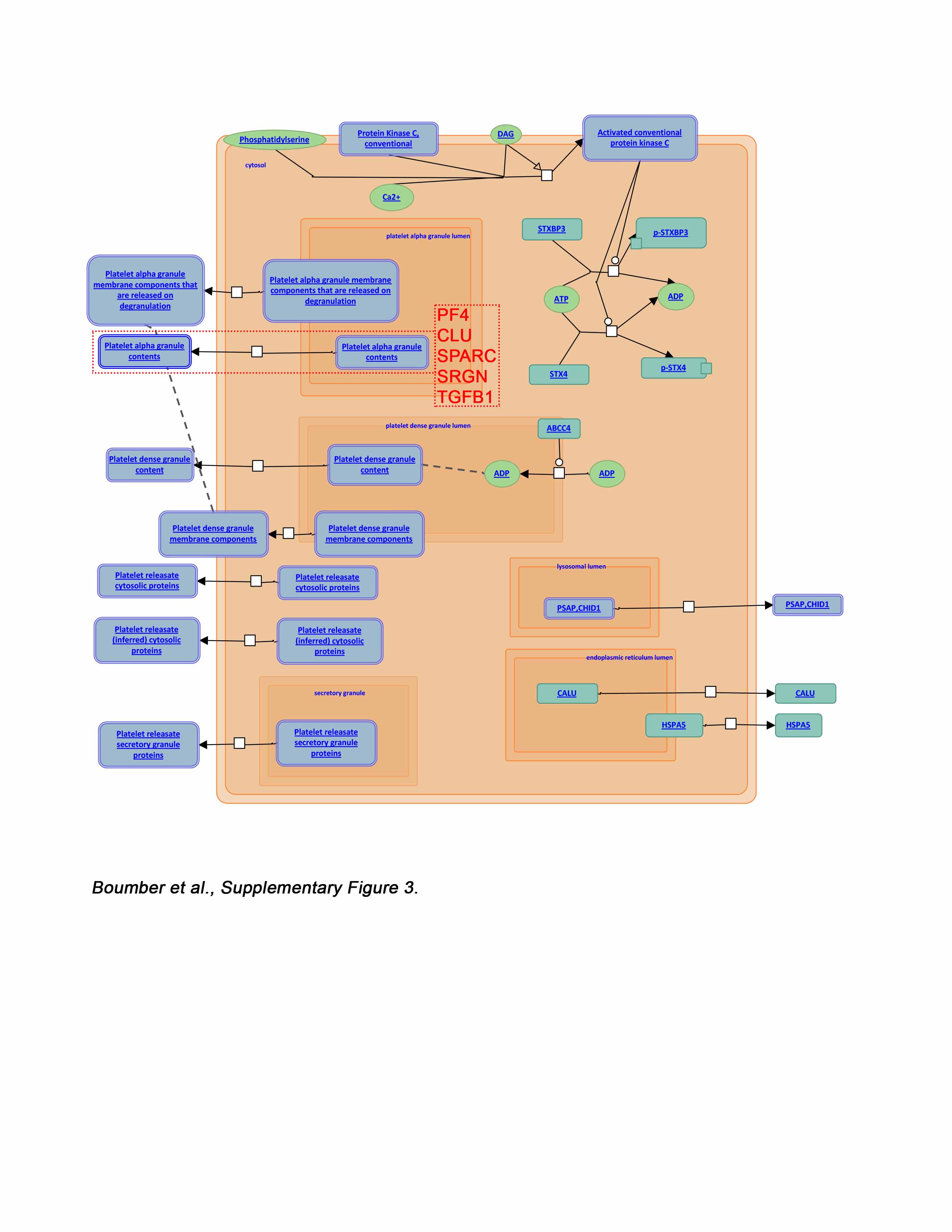

Supplement: Supplementary file 3 — Figure S3. Platelet degranulation pathway. Platelet alpha granule contents includes three out of four (CLU, SPARC, SRGN) identified genes as well as PF4 and TGFB1. The figure was generated using Reactome. (JPG 348 kb) [file 12885_2019_5795_MOESM3_ESM.jpg]
