# Supplementary material for: Circulating tumor cell and cell-free RNA capture and expression analysis identify platelet-associated genes in metastatic lung cancer
Source: BMC Cancer. 2019 Jun 19;19:603. doi: 10.1186/s12885-019-5795-x (PMC6582501; doi:10.1186/s12885-019-5795-x)
Supplement: Supplementary file 4 — Table S1. List of 770 screened genes. (DOCX 54 kb) [file 12885_2019_5795_MOESM4_ESM.docx]

**Table S1. List of 770 screened genes.**

| **Type** | **Gene Name** | **RefSeq #** |
| --- | --- | --- |
| Endogenous | AAMP | NM_001087.3 |
| Endogenous | ABI3BP | NM_015429.3 |
| Endogenous | ACHE | NM_000665.3 |
| Endogenous | ACTG2 | NM_001615.3 |
| Endogenous | ACVR1 | NM_001105.2 |
| Endogenous | ACVR1C | NM_145259.2 |
| Endogenous | ACVRL1 | NM_000020.1 |
| Endogenous | ADAM15 | NM_207195.1 |
| Endogenous | ADAM17 | NM_003183.4 |
| Endogenous | ADAM28 | NM_014265.4 |
| Endogenous | ADAM8 | NM_001109.4 |
| Endogenous | ADAM9 | NM_001005845.1 |
| Endogenous | ADAMTS1 | NM_006988.3 |
| Endogenous | ADAMTS12 | NM_030955.2 |
| Endogenous | ADAMTS8 | NM_007037.4 |
| Endogenous | ADAP1 | NM_006869.2 |
| Endogenous | ADD1 | NM_001119.4 |
| Endogenous | ADM2 | NM_001253845.1 |
| Endogenous | ADRA2B | NM_000682.4 |
| Endogenous | AEBP1 | NM_001129.3 |
| Endogenous | AGGF1 | NM_018046.3 |
| Endogenous | AGR2 | NM_006408.3 |
| Endogenous | AGRN | NM_198576.2 |
| Endogenous | AGT | NM_000029.3 |
| Endogenous | AHNAK | NM_001620.2 |
| Endogenous | AKAP12 | NM_005100.3 |
| Endogenous | AKAP2 | NM_001004065.4 |
| Endogenous | AKT1 | NM_005163.2 |
| Endogenous | AKT2 | NM_001626.2 |
| Endogenous | AKT3 | NM_005465.4 |
| Endogenous | ALB | NM_000477.5 |
| Endogenous | ALDOA | NM_184041.2 |
| Endogenous | ALOX5 | NM_000698.2 |
| Endogenous | AMH | NM_000479.3 |
| Endogenous | ANG | NM_001145.4 |
| Endogenous | ANGPT1 | NM_001146.3 |
| Endogenous | ANGPT2 | NM_001147.2 |
| Endogenous | ANGPTL2 | NM_012098.2 |
| Endogenous | ANGPTL4 | NR_104213.1 |
| Endogenous | ANPEP | NM_001150.1 |
| Endogenous | ANXA2P2 | NR_003573.1 |
| Endogenous | AP1M2 | NM_005498.4 |
| Endogenous | APC | NM_000038.3 |
| Endogenous | APOD | NM_001647.3 |
| Endogenous | APOE | NM_000041.2 |
| Endogenous | APOH | NM_000042.2 |
| Endogenous | AQP1 | NM_198098.1 |
| Endogenous | ARAP2 | NM_015230.2 |
| Endogenous | AREG | NM_001657.2 |
| Endogenous | ARHGAP32 | NM_001142685.1 |
| Endogenous | ARHGDIB | NM_001175.4 |
| Endogenous | ASPN | NM_017680.3 |
| Endogenous | ATPIF1 | NM_178190.2 |
| Endogenous | B3GNT3 | NM_014256.3 |
| Endogenous | BAD | NM_004322.3 |
| Endogenous | BAG2 | NM_004282.3 |
| Endogenous | BAI1 | NM_001702.1 |
| Endogenous | BAI3 | NM_001704.1 |
| Endogenous | BCAS1 | NM_003657.2 |
| Endogenous | BGN | NM_001711.3 |
| Endogenous | BICC1 | NM_001080512.1 |
| Endogenous | BMP4 | NM_001202.3 |
| Endogenous | BMP5 | NM_021073.2 |
| Endogenous | BMP7 | NM_001719.2 |
| Endogenous | BMPER | NM_133468.4 |
| Endogenous | BMPR1A | NM_004329.2 |
| Endogenous | BMPR1B | NM_001203.1 |
| Endogenous | BMPR2 | NM_001204.5 |
| Endogenous | BNC2 | NM_017637.5 |
| Endogenous | BRMS1 | NM_015399.3 |
| Endogenous | BTG1 | NM_001731.2 |
| Endogenous | C1S | NM_001734.2 |
| Endogenous | C3 | NM_000064.2 |
| Endogenous | C3AR1 | NM_004054.2 |
| Endogenous | CADM1 | NM_014333.3 |
| Endogenous | CALCRL | NM_005795.3 |
| Endogenous | CALD1 | NM_004342.6 |
| Endogenous | CAMK2A | NM_171825.1 |
| Endogenous | CAMK2B | NM_001220.3 |
| Endogenous | CAMK2D | NM_172127.1 |
| Endogenous | CAMP | NM_004345.3 |
| Endogenous | CASP8 | NM_001228.4 |
| Endogenous | CAV1 | NM_001753.3 |
| Endogenous | CBLC | NM_012116.3 |
| Endogenous | CCBE1 | NM_133459.3 |
| Endogenous | CCDC80 | NM_199511.1 |
| Endogenous | CCL11 | NM_002986.2 |
| Endogenous | CCL21 | NM_002989.2 |
| Endogenous | CCL5 | NM_002985.2 |
| Endogenous | CCL7 | NM_006273.2 |
| Endogenous | CCL8 | NM_005623.2 |
| Endogenous | CCR2 | NM_001123041.2 |
| Endogenous | CCR3 | NM_001837.2 |
| Endogenous | CD163 | NM_004244.4 |
| Endogenous | CD24 | NM_013230.2 |
| Endogenous | CD2AP | NM_012120.2 |
| Endogenous | CD34 | NM_001773.2 |
| Endogenous | CD36 | NM_000072.3 |
| Endogenous | CD44 | NM_001001392.1 |
| Endogenous | CD46 | NM_172350.1 |
| Endogenous | CD82 | NM_002231.3 |
| Endogenous | CDC42 | NM_001039802.1 |
| Endogenous | CDH1 | NM_004360.2 |
| Endogenous | CDH11 | NM_001797.2 |
| Endogenous | CDH13 | NM_001220488.1 |
| Endogenous | CDH2 | NM_001792.3 |
| Endogenous | CDK14 | NM_012395.2 |
| Endogenous | CDKN1A | NM_000389.2 |
| Endogenous | CDKN2A | NM_000077.3 |
| Endogenous | CDS1 | NM_001263.3 |
| Endogenous | CEACAM1 | NM_001712.3 |
| Endogenous | CEACAM5 | NM_004363.2 |
| Endogenous | CEACAM6 | NM_002483.4 |
| Endogenous | CEP170 | NM_001042404.1 |
| Endogenous | CEP295 | NM_033395.1 |
| Endogenous | CFP | NM_002621.2 |
| Endogenous | CGN | NM_020770.2 |
| Endogenous | CHAD | NM_001267.2 |
| Endogenous | CHD4 | NM_001273.2 |
| Endogenous | CHI3L1 | NM_001276.2 |
| Endogenous | CHP1 | XM_005254140.1 |
| Endogenous | CHP2 | NM_022097.3 |
| Endogenous | CHRDL1 | NM_001143981.1 |
| Endogenous | CHRNA7 | NR_046324.1 |
| Endogenous | CIB1 | NM_001277764.1 |
| Endogenous | CKMT1A | NM_001015001.1 |
| Endogenous | CLDN1 | NM_021101.3 |
| Endogenous | CLDN3 | NM_001306.3 |
| Endogenous | CLDN4 | NM_001305.3 |
| Endogenous | CLDN7 | NM_001307.3 |
| Endogenous | CLEC2B | NM_005127.2 |
| Endogenous | CLEC3B | NM_003278.2 |
| Endogenous | CLIC4 | NM_013943.2 |
| Endogenous | CLU | NM_203339.2 |
| Endogenous | CMA1 | NM_001836.2 |
| Endogenous | CNN1 | NM_001299.4 |
| Endogenous | COL18A1 | NM_030582.3 |
| Endogenous | COL1A1 | NM_000088.3 |
| Endogenous | COL1A2 | NM_000089.3 |
| Endogenous | COL3A1 | NM_000090.3 |
| Endogenous | COL4A1 | NM_001845.4 |
| Endogenous | COL4A2 | NM_001846.2 |
| Endogenous | COL4A6 | NM_001847.2 |
| Endogenous | COL5A1 | NM_000093.3 |
| Endogenous | COL5A2 | NM_000393.3 |
| Endogenous | COL6A1 | NM_001848.2 |
| Endogenous | COL6A2 | NM_001849.2 |
| Endogenous | COL6A3 | NM_004369.3 |
| Endogenous | COL7A1 | NM_000094.2 |
| Endogenous | COMP | NM_000095.2 |
| Endogenous | CREBBP | NM_001079846.1 |
| Endogenous | CRIP2 | NM_001270837.1 |
| Endogenous | CRISPLD2 | NM_031476.3 |
| Endogenous | CSF2RB | NM_000395.2 |
| Endogenous | CSPG4 | NM_001897.4 |
| Endogenous | CST7 | NM_003650.3 |
| Endogenous | CTNNB1 | NM_001098210.1 |
| Endogenous | CTNND1 | NM_001331.2 |
| Endogenous | CTSG | NM_001911.2 |
| Endogenous | CTSH | NM_148979.2 |
| Endogenous | CTSK | NM_000396.2 |
| Endogenous | CTSL | NM_001912.4 |
| Endogenous | CUL1 | NM_003592.2 |
| Endogenous | CX3CL1 | NM_002996.3 |
| Endogenous | CXADR | NM_001338.3 |
| Endogenous | CXCL10 | NM_001565.1 |
| Endogenous | CXCL11 | NM_005409.3 |
| Endogenous | CXCL12 | NM_199168.3 |
| Endogenous | CXCL13 | NM_006419.2 |
| Endogenous | CXCL17 | NM_198477.1 |
| Endogenous | CXCL8 | NM_000584.2 |
| Endogenous | CXCR2 | NM_001168298.1 |
| Endogenous | CXCR3 | NM_001504.1 |
| Endogenous | CXCR4 | NM_003467.2 |
| Endogenous | CYB561 | NM_001915.3 |
| Endogenous | CYBB | NM_000397.3 |
| Endogenous | CYP1B1 | NM_000104.3 |
| Endogenous | DAG1 | NM_001165928.2 |
| Endogenous | DCC | NM_005215.1 |
| Endogenous | DCN | NM_001920.3 |
| Endogenous | DDR2 | NM_006182.2 |
| Endogenous | DENND5A | NM_015213.2 |
| Endogenous | DENR | NM_003677.3 |
| Endogenous | DESI1 | NM_015704.2 |
| Endogenous | DICER1 | NM_177438.2 |
| Endogenous | DLC1 | NM_006094.3 |
| Endogenous | DLG1 | NM_001098424.1 |
| Endogenous | DLL4 | NM_019074.2 |
| Endogenous | DPT | NM_001937.3 |
| Endogenous | DPYSL3 | NM_001387.2 |
| Endogenous | DSC2 | NM_024422.3 |
| Endogenous | DST | NM_001723.4 |
| Endogenous | ECM1 | NM_004425.3 |
| Endogenous | ECM2 | NM_001393.2 |
| Endogenous | ECSCR | NM_001077693.3 |
| Endogenous | EDN1 | NM_001955.2 |
| Endogenous | EGF | NM_001963.4 |
| Endogenous | EGFL7 | NM_016215.3 |
| Endogenous | EGFR | NM_201282.1 |
| Endogenous | EGLN2 | NM_053046.3 |
| Endogenous | EGLN3 | NM_022073.3 |
| Endogenous | EIF2AK3 | NM_004836.5 |
| Endogenous | EIF4E2 | NM_004846.3 |
| Endogenous | EIF4EBP1 | NM_004095.3 |
| Endogenous | ELF3 | NM_001114309.1 |
| Endogenous | ELK3 | NM_005230.2 |
| Endogenous | EMCN | NM_016242.3 |
| Endogenous | EMILIN1 | XM_006711928.1 |
| Endogenous | EMILIN3 | NM_052846.1 |
| Endogenous | EMP3 | NM_001425.2 |
| Endogenous | ENO1 | NM_001428.2 |
| Endogenous | ENO2 | NM_001975.2 |
| Endogenous | ENO3 | NM_001976.4 |
| Endogenous | ENPEP | NM_001977.3 |
| Endogenous | ENPP2 | NM_001040092.2 |
| Endogenous | EP300 | NM_001429.2 |
| Endogenous | EPAS1 | NM_001430.3 |
| Endogenous | EPCAM | NM_002354.1 |
| Endogenous | EPHA1 | NM_005232.3 |
| Endogenous | EPHA2 | NM_004431.2 |
| Endogenous | EPHB1 | NM_004441.3 |
| Endogenous | EPHB3 | NM_004443.3 |
| Endogenous | EPHB4 | NM_004444.4 |
| Endogenous | EPN3 | NM_017957.2 |
| Endogenous | EPS8L1 | NM_017729.3 |
| Endogenous | ERBB2 | NM_001005862.1 |
| Endogenous | ERBB2IP | NM_018695.2 |
| Endogenous | ERBB3 | NM_001005915.1 |
| Endogenous | EREG | NM_001432.2 |
| Endogenous | ERMP1 | NM_024896.2 |
| Endogenous | ESRP1 | NM_001034915.2 |
| Endogenous | ETV4 | NM_001079675.1 |
| Endogenous | EVI2A | NM_014210.3 |
| Endogenous | EVPL | NM_001988.2 |
| Endogenous | F11R | NM_144503.1 |
| Endogenous | F3 | NM_001993.3 |
| Endogenous | FAM174B | NM_207446.2 |
| Endogenous | FAP | NM_004460.2 |
| Endogenous | FASLG | NM_000639.1 |
| Endogenous | FBLN1 | NM_006487.2 |
| Endogenous | FBLN5 | NM_006329.3 |
| Endogenous | FBN1 | NM_000138.3 |
| Endogenous | FBN2 | NM_001999.3 |
| Endogenous | FBP1 | NM_000507.3 |
| Endogenous | FERMT2 | NM_001135000.1 |
| Endogenous | FGF18 | NM_003862.1 |
| Endogenous | FGF2 | NM_002006.4 |
| Endogenous | FGF9 | NM_002010.2 |
| Endogenous | FGFR1 | NM_015850.2 |
| Endogenous | FGFR2 | NM_000141.4 |
| Endogenous | FGFR3 | NM_022965.2 |
| Endogenous | FGFR4 | NM_002011.3 |
| Endogenous | FGL2 | NM_006682.2 |
| Endogenous | FHL1 | NM_001449.4 |
| Endogenous | FIGF | NM_004469.2 |
| Endogenous | FLI1 | NM_001167681.2 |
| Endogenous | FLT1 | NM_002019.4 |
| Endogenous | FLT4 | NM_002020.1 |
| Endogenous | FMOD | NM_002023.3 |
| Endogenous | FN1 | NM_212482.1 |
| Endogenous | FOXC2 | NM_005251.2 |
| Endogenous | FOXO4 | NM_005938.2 |
| Endogenous | FRAS1 | NM_001166133.1 |
| Endogenous | FREM1 | NM_001177704.1 |
| Endogenous | FREM2 | NM_207361.4 |
| Endogenous | FST | NM_006350.2 |
| Endogenous | FSTL1 | NM_007085.4 |
| Endogenous | FUT3 | NM_000149.3 |
| Endogenous | FXYD6 | NM_001164831.1 |
| Endogenous | GALNT7 | NM_017423.2 |
| Endogenous | GATA4 | NM_002052.3 |
| Endogenous | GDF15 | NM_004864.2 |
| Endogenous | GDF5 | NM_000557.2 |
| Endogenous | GDF6 | NM_001001557.2 |
| Endogenous | GIMAP4 | NM_018326.2 |
| Endogenous | GIMAP6 | NR_024115.1 |
| Endogenous | GJA5 | NM_005266.5 |
| Endogenous | GLYR1 | NM_032569.3 |
| Endogenous | GPI | NM_000175.2 |
| Endogenous | GPR124 | NM_032777.9 |
| Endogenous | GPR56 | NM_005682.4 |
| Endogenous | GPX1 | NM_000581.2 |
| Endogenous | GREM1 | NM_013372.5 |
| Endogenous | GRHL2 | NM_024915.3 |
| Endogenous | GSN | NM_000177.4 |
| Endogenous | GTF2I | NM_033001.2 |
| Endogenous | GZMK | NM_002104.2 |
| Endogenous | HAPLN1 | NM_001884.3 |
| Endogenous | HAS1 | NM_001523.2 |
| Endogenous | HDAC5 | NM_005474.4 |
| Endogenous | HDHD3 | NM_031219.2 |
| Endogenous | HEG1 | NM_020733.1 |
| Endogenous | HGF | NM_000601.4 |
| Endogenous | HIF1A | NM_001530.2 |
| Endogenous | HIPK1 | NM_152696.3 |
| Endogenous | HIPK2 | NM_022740.4 |
| Endogenous | HK2 | NM_000189.4 |
| Endogenous | HK3 | NM_002115.1 |
| Endogenous | HKDC1 | NM_025130.3 |
| Endogenous | HLA-DPB1 | NM_002121.4 |
| Endogenous | HMOX1 | NM_002133.2 |
| Endogenous | HOXA5 | NM_019102.2 |
| Endogenous | HOXA7 | NM_006896.3 |
| Endogenous | HOXB13 | NM_006361.5 |
| Endogenous | HOXB3 | NM_002146.4 |
| Endogenous | HPSE | NM_006665.3 |
| Endogenous | HRAS | NM_005343.2 |
| Endogenous | HSD17B12 | NM_016142.2 |
| Endogenous | HSP90B1 | NM_003299.1 |
| Endogenous | HSPB1 | NM_001540.3 |
| Endogenous | HSPG2 | NM_005529.5 |
| Endogenous | HUNK | NM_014586.1 |
| Endogenous | IBSP | NM_004967.3 |
| Endogenous | ICAM1 | NM_000201.2 |
| Endogenous | ID1 | NM_002165.2 |
| Endogenous | ID2 | NM_002166.4 |
| Endogenous | ID4 | NM_001546.2 |
| Endogenous | IFNG | NM_000619.2 |
| Endogenous | IGF1 | NM_000618.3 |
| Endogenous | IGFBP4 | NM_001552.2 |
| Endogenous | IGFBP7 | NM_001553.1 |
| Endogenous | IL10RA | NM_001558.2 |
| Endogenous | IL11 | NM_000641.2 |
| Endogenous | IL13RA2 | NM_000640.2 |
| Endogenous | IL15 | NM_172174.1 |
| Endogenous | IL18 | NM_001562.2 |
| Endogenous | IL1A | NM_000575.3 |
| Endogenous | IL1B | NM_000576.2 |
| Endogenous | IL1RL1 | NM_016232.4 |
| Endogenous | IL1RN | NM_000577.3 |
| Endogenous | IL6 | NM_000600.1 |
| Endogenous | ILK | NM_004517.2 |
| Endogenous | INHBA | NM_002192.2 |
| Endogenous | INHBE | NM_031479.3 |
| Endogenous | IRF6 | NM_006147.2 |
| Endogenous | ISL1 | NM_002202.2 |
| Endogenous | ISLR | NM_005545.3 |
| Endogenous | ITGA1 | NM_181501.1 |
| Endogenous | ITGA11 | NM_012211.3 |
| Endogenous | ITGA2 | NM_002203.2 |
| Endogenous | ITGA3 | NM_002204.2 |
| Endogenous | ITGA5 | NM_002205.2 |
| Endogenous | ITGA6 | NM_000210.1 |
| Endogenous | ITGA7 | NM_002206.1 |
| Endogenous | ITGA8 | NM_003638.1 |
| Endogenous | ITGA9 | NM_002207.2 |
| Endogenous | ITGAM | NM_000632.3 |
| Endogenous | ITGB1 | NM_033666.2 |
| Endogenous | ITGB1BP1 | NM_004763.3 |
| Endogenous | ITGB2 | NM_000211.2 |
| Endogenous | ITGB3 | NM_000212.2 |
| Endogenous | ITGB4 | NM_001005731.1 |
| Endogenous | ITGB6 | NM_000888.3 |
| Endogenous | ITGB7 | NM_000889.1 |
| Endogenous | ITGB8 | NM_002214.2 |
| Endogenous | ITM2A | NM_004867.4 |
| Endogenous | JAG1 | NM_000214.2 |
| Endogenous | JAM2 | NM_001270407.1 |
| Endogenous | JAM3 | NM_032801.3 |
| Endogenous | JUN | NM_002228.3 |
| Endogenous | KCNJ8 | NM_004982.2 |
| Endogenous | KDM1A | NM_015013.3 |
| Endogenous | KDR | NM_002253.2 |
| Endogenous | KIAA1462 | NM_020848.2 |
| Endogenous | KISS1 | NM_002256.3 |
| Endogenous | KLK3 | NM_001030049.1 |
| Endogenous | KRAS | NM_004985.3 |
| Endogenous | KRIT1 | NM_004912.3 |
| Endogenous | KRT1 | NM_006121.2 |
| Endogenous | KRT14 | NM_000526.4 |
| Endogenous | KRT19 | NM_002276.4 |
| Endogenous | KRT7 | NM_005556.3 |
| Endogenous | LAD1 | NM_005558.3 |
| Endogenous | LAMA1 | NM_005559.2 |
| Endogenous | LAMA3 | NM_000227.3 |
| Endogenous | LAMA4 | NM_001105209.1 |
| Endogenous | LAMA5 | NM_005560.3 |
| Endogenous | LAMB3 | NM_000228.2 |
| Endogenous | LAMC1 | NM_002293.3 |
| Endogenous | LAMC2 | NM_005562.2 |
| Endogenous | LDHA | NM_001165414.1 |
| Endogenous | LEFTY1 | NM_020997.2 |
| Endogenous | LGALS1 | NM_002305.3 |
| Endogenous | LHFP | NM_005780.2 |
| Endogenous | LIFR | NM_002310.3 |
| Endogenous | LLGL2 | NM_001015002.1 |
| Endogenous | LOX | NM_002317.4 |
| Endogenous | LOXL2 | NM_002318.2 |
| Endogenous | LRG1 | NM_052972.2 |
| Endogenous | LTBP4 | NM_003573.2 |
| Endogenous | LUM | NM_002345.3 |
| Endogenous | LY96 | NM_015364.2 |
| Endogenous | MAF | NM_005360.4 |
| Endogenous | MAP2K1 | NM_002755.2 |
| Endogenous | MAP2K2 | NM_030662.3 |
| Endogenous | MAP2K4 | NM_003010.2 |
| Endogenous | MAP3K7 | NM_145333.1 |
| Endogenous | MAPK1 | NM_138957.2 |
| Endogenous | MAPK3 | NM_001040056.1 |
| Endogenous | MAPKAPK3 | NM_004635.3 |
| Endogenous | MCAM | NM_006500.2 |
| Endogenous | MED1 | NM_004774.3 |
| Endogenous | MED23 | NM_004830.2 |
| Endogenous | MEG3 | NR_002766.2 |
| Endogenous | MEOX2 | NM_005924.4 |
| Endogenous | MET | NM_001127500.1 |
| Endogenous | MFAP4 | NM_002404.1 |
| Endogenous | MGAT5 | NM_002410.4 |
| Endogenous | MGP | NM_000900.2 |
| Endogenous | MISP | NM_173481.2 |
| Endogenous | MMP1 | NM_002421.2 |
| Endogenous | MMP10 | NM_002425.1 |
| Endogenous | MMP12 | NM_002426.3 |
| Endogenous | MMP13 | NM_002427.2 |
| Endogenous | MMP14 | NM_004995.2 |
| Endogenous | MMP17 | NM_016155.4 |
| Endogenous | MMP2 | NM_004530.2 |
| Endogenous | MMP24 | NM_006690.3 |
| Endogenous | MMP3 | NM_002422.3 |
| Endogenous | MMP9 | NM_004994.2 |
| Endogenous | MMRN2 | NM_024756.2 |
| Endogenous | MPDZ | NM_003829.4 |
| Endogenous | MRC1 | NM_002438.2 |
| Endogenous | MS4A4A | NM_024021.2 |
| Endogenous | MS4A6A | NM_152852.2 |
| Endogenous | MT3 | NM_005954.2 |
| Endogenous | MTA1 | NM_004689.2 |
| Endogenous | MTBP | NM_022045.4 |
| Endogenous | MTDH | NM_178812.3 |
| Endogenous | MTOR | NM_004958.2 |
| Endogenous | MUC1 | NM_001018017.1 |
| Endogenous | MYC | NM_002467.3 |
| Endogenous | MYCL | NM_001033081.2 |
| Endogenous | MYH11 | NM_001040113.1 |
| Endogenous | MYLK | NM_053032.2 |
| Endogenous | MYO1D | NM_015194.1 |
| Endogenous | MYO5C | NM_018728.2 |
| Endogenous | NAA15 | NM_057175.3 |
| Endogenous | NAP1L3 | NM_004538.4 |
| Endogenous | NCAM1 | NM_000615.5 |
| Endogenous | NCL | NM_005381.2 |
| Endogenous | NDNF | NM_024574.3 |
| Endogenous | NDP | NM_000266.2 |
| Endogenous | NDRG1 | NM_006096.2 |
| Endogenous | NF1 | NM_000267.2 |
| Endogenous | NF2 | NM_181825.2 |
| Endogenous | NFAT5 | NM_173214.1 |
| Endogenous | NFATC2 | NM_012340.3 |
| Endogenous | NFKB1 | NM_003998.2 |
| Endogenous | NID2 | NM_007361.3 |
| Endogenous | NME1 | NM_000269.2 |
| Endogenous | NME4 | NM_005009.2 |
| Endogenous | NODAL | NM_018055.3 |
| Endogenous | NOS2 | NM_153292.1 |
| Endogenous | NOS3 | NM_000603.4 |
| Endogenous | NOTCH1 | NM_017617.3 |
| Endogenous | NOX5 | NM_024505.2 |
| Endogenous | NPR1 | NM_000906.2 |
| Endogenous | NR3C1 | NM_001018077.1 |
| Endogenous | NR4A1 | NM_173157.1 |
| Endogenous | NR4A3 | NM_173198.1 |
| Endogenous | NRCAM | NM_005010.4 |
| Endogenous | NRP1 | NM_003873.5 |
| Endogenous | NRP2 | NM_003872.2 |
| Endogenous | NRXN1 | NM_138735.2 |
| Endogenous | NRXN3 | NM_001105250.1 |
| Endogenous | NTRK1 | NM_001012331.1 |
| Endogenous | OAS1 | NM_001032409.1 |
| Endogenous | OCLN | NM_002538.3 |
| Endogenous | OGN | NM_014057.3 |
| Endogenous | OLFML2B | NM_015441.1 |
| Endogenous | OVOL2 | NM_021220.2 |
| Endogenous | P3H1 | NM_001146289.1 |
| Endogenous | P3H2 | NM_018192.2 |
| Endogenous | PCOLCE | NM_002593.3 |
| Endogenous | PDCD10 | NM_145859.1 |
| Endogenous | PDCL3 | NM_024065.4 |
| Endogenous | PDGFA | NM_002607.5 |
| Endogenous | PDGFC | NM_016205.2 |
| Endogenous | PDGFRB | NM_002609.3 |
| Endogenous | PDK1 | NM_002610.3 |
| Endogenous | PDPN | NM_006474.4 |
| Endogenous | PEBP4 | NM_144962.2 |
| Endogenous | PECAM1 | NM_000442.3 |
| Endogenous | PFKFB1 | NM_002625.2 |
| Endogenous | PFKFB4 | NM_004567.2 |
| Endogenous | PGK1 | NM_000291.2 |
| Endogenous | PIK3CA | NM_006218.2 |
| Endogenous | PIK3CD | NM_005026.3 |
| Endogenous | PIK3CG | NM_002649.2 |
| Endogenous | PIK3R1 | NM_181504.2 |
| Endogenous | PIK3R2 | NM_005027.2 |
| Endogenous | PIK3R5 | NM_001142633.1 |
| Endogenous | PIK3R6 | NM_001010855.3 |
| Endogenous | PITX2 | NM_000325.5 |
| Endogenous | PKM | NM_182471.1 |
| Endogenous | PKN1 | NM_213560.1 |
| Endogenous | PKNOX1 | NM_004571.3 |
| Endogenous | PLA2G10 | NM_003561.1 |
| Endogenous | PLA2G2A | NM_000300.2 |
| Endogenous | PLA2G2D | NM_001271814.1 |
| Endogenous | PLA2G3 | NM_015715.3 |
| Endogenous | PLAU | NM_002658.2 |
| Endogenous | PLAUR | NM_001005376.1 |
| Endogenous | PLCG1 | NM_002660.2 |
| Endogenous | PLCG2 | NM_002661.2 |
| Endogenous | PLEKHO1 | NM_016274.4 |
| Endogenous | PLS1 | NM_002670.2 |
| Endogenous | PLXDC1 | NM_020405.4 |
| Endogenous | PLXNC1 | NM_005761.2 |
| Endogenous | PLXND1 | NM_015103.2 |
| Endogenous | PMP22 | NM_000304.2 |
| Endogenous | PNPLA6 | NM_006702.3 |
| Endogenous | POPDC3 | NM_022361.4 |
| Endogenous | POSTN | NM_001135935.1 |
| Endogenous | PPFIBP2 | NM_003621.2 |
| Endogenous | PPL | NM_002705.4 |
| Endogenous | PPP1R16B | NM_015568.2 |
| Endogenous | PPP2CB | NM_001009552.1 |
| Endogenous | PPP2R1A | NM_014225.3 |
| Endogenous | PPP3R1 | NM_000945.3 |
| Endogenous | PRELP | NM_002725.3 |
| Endogenous | PRF1 | NM_005041.3 |
| Endogenous | PRKCB | NM_212535.1 |
| Endogenous | PRKCG | NM_002739.3 |
| Endogenous | PRKCZ | NM_002744.4 |
| Endogenous | PROK2 | NM_021935.3 |
| Endogenous | PROM1 | NM_006017.1 |
| Endogenous | PRR15L | NM_024320.2 |
| Endogenous | PRSS22 | NM_022119.3 |
| Endogenous | PRSS8 | NM_002773.3 |
| Endogenous | PTEN | NM_000314.4 |
| Endogenous | PTGDS | NM_000954.5 |
| Endogenous | PTGIS | NM_000961.3 |
| Endogenous | PTGS2 | NM_000963.1 |
| Endogenous | PTK2 | NM_153831.2 |
| Endogenous | PTK2B | NM_004103.3 |
| Endogenous | PTK6 | NM_005975.2 |
| Endogenous | PTPRB | NM_002837.3 |
| Endogenous | PTPRC | NM_080923.2 |
| Endogenous | PTPRM | NM_002845.3 |
| Endogenous | PTRF | NM_012232.5 |
| Endogenous | PTTG1 | NM_004219.2 |
| Endogenous | PTX3 | NM_002852.3 |
| Endogenous | PXDN | NM_012293.1 |
| Endogenous | PYCARD | NM_013258.3 |
| Endogenous | QKI | NM_006775.2 |
| Endogenous | RAB25 | NM_020387.2 |
| Endogenous | RAC1 | NM_198829.1 |
| Endogenous | RAC2 | NM_002872.3 |
| Endogenous | RAF1 | NM_002880.3 |
| Endogenous | RAMP1 | NM_005855.2 |
| Endogenous | RAMP2 | NM_005854.2 |
| Endogenous | RB1 | NM_000321.1 |
| Endogenous | RBL1 | NM_183404.1 |
| Endogenous | RBL2 | NM_005611.3 |
| Endogenous | RBM47 | NM_019027.3 |
| Endogenous | RBPJ | NM_015874.3 |
| Endogenous | RBX1 | NM_014248.2 |
| Endogenous | RELN | NM_005045.2 |
| Endogenous | RGCC | NM_014059.2 |
| Endogenous | RHOA | NM_001664.2 |
| Endogenous | RNH1 | NM_203384.1 |
| Endogenous | ROBO4 | NM_019055.5 |
| Endogenous | ROCK1 | NM_005406.1 |
| Endogenous | ROCK2 | NM_004850.3 |
| Endogenous | RORA | NM_134261.2 |
| Endogenous | RORB | NM_006914.3 |
| Endogenous | RPS27A | NM_002954.5 |
| Endogenous | RPS6KB1 | NM_003161.2 |
| Endogenous | RPS6KB2 | NM_003952.2 |
| Endogenous | RRAS | NM_006270.3 |
| Endogenous | RTN4 | NM_007008.2 |
| Endogenous | RUNX1 | NM_001754.4 |
| Endogenous | RUNX1T1 | NM_004349.2 |
| Endogenous | S100A14 | NM_020672.1 |
| Endogenous | S100A7 | NM_002963.2 |
| Endogenous | S1PR1 | NM_001400.4 |
| Endogenous | SACS | NM_014363.4 |
| Endogenous | SAMSN1 | NM_022136.3 |
| Endogenous | SCG2 | NM_003469.3 |
| Endogenous | SCNN1A | NM_001038.4 |
| Endogenous | SDC4 | NM_002999.2 |
| Endogenous | SELE | NM_000450.2 |
| Endogenous | SEMA3E | NM_012431.1 |
| Endogenous | SERINC5 | NM_001174071.1 |
| Endogenous | SERPINA1 | NM_000295.4 |
| Endogenous | SERPINE1 | NM_001165413.1 |
| Endogenous | SERPINF1 | NM_002615.4 |
| Endogenous | SERPING1 | NM_000062.2 |
| Endogenous | SERPINH1 | NM_001235.2 |
| Endogenous | SET | NM_001122821.1 |
| Endogenous | SETD2 | NM_014159.6 |
| Endogenous | SFRP1 | NM_003012.3 |
| Endogenous | SFRP2 | NM_003013.2 |
| Endogenous | SH2B3 | NM_005475.2 |
| Endogenous | SH2D3A | NM_005490.2 |
| Endogenous | SH3YL1 | NM_001159597.1 |
| Endogenous | SHB | NM_003028.2 |
| Endogenous | SIRT1 | NM_012238.4 |
| Endogenous | SKP1 | NM_170679.2 |
| Endogenous | SLC12A6 | NM_001042494.1 |
| Endogenous | SLC2A1 | NM_006516.2 |
| Endogenous | SLC35A3 | NM_012243.1 |
| Endogenous | SLC37A1 | NM_018964.3 |
| Endogenous | SLC44A4 | NM_032794.1 |
| Endogenous | SLIT2 | NM_004787.1 |
| Endogenous | SLPI | NM_003064.2 |
| Endogenous | SMAD1 | NM_005900.2 |
| Endogenous | SMAD2 | NM_005901.5 |
| Endogenous | SMAD3 | NM_005902.3 |
| Endogenous | SMAD4 | NM_005359.3 |
| Endogenous | SMAD5 | NM_005903.5 |
| Endogenous | SMAD9 | NM_001127217.2 |
| Endogenous | SMC3 | NM_005445.3 |
| Endogenous | SMOC1 | NM_001034852.1 |
| Endogenous | SMURF1 | NM_181349.1 |
| Endogenous | SMURF2 | NM_022739.3 |
| Endogenous | SNAI1 | NM_005985.2 |
| Endogenous | SNAI2 | NM_003068.3 |
| Endogenous | SNAI3 | NM_178310.1 |
| Endogenous | SNRPF | NM_003095.2 |
| Endogenous | SOD1 | NM_000454.4 |
| Endogenous | SORD | NM_003104.4 |
| Endogenous | SOX17 | NM_022454.3 |
| Endogenous | SOX2 | NM_003106.2 |
| Endogenous | SOX9 | NM_000346.2 |
| Endogenous | SP1 | NM_003109.1 |
| Endogenous | SPARC | NM_003118.2 |
| Endogenous | SPARCL1 | NM_004684.4 |
| Endogenous | SPDEF | NM_012391.1 |
| Endogenous | SPHK2 | NM_020126.3 |
| Endogenous | SPINK5 | NM_006846.3 |
| Endogenous | SPINT1 | NM_001032367.1 |
| Endogenous | SPOCK3 | NM_001204355.1 |
| Endogenous | SPP1 | NM_000582.2 |
| Endogenous | SRC | NM_005417.3 |
| Endogenous | SRF | NM_003131.2 |
| Endogenous | SRGN | NR_036430.1 |
| Endogenous | SRPK2 | NM_182692.1 |
| Endogenous | SRPX2 | NM_014467.2 |
| Endogenous | SSTR2 | NM_001050.2 |
| Endogenous | ST14 | NM_021978.3 |
| Endogenous | STAB1 | NM_015136.2 |
| Endogenous | STAB2 | NM_017564.9 |
| Endogenous | STAT1 | NM_139266.1 |
| Endogenous | STAT3 | NM_139276.2 |
| Endogenous | SULF1 | NM_001128204.1 |
| Endogenous | SV2B | NM_001167580.1 |
| Endogenous | SYK | NM_003177.3 |
| Endogenous | SYNE1 | NM_015293.1 |
| Endogenous | TACSTD2 | NM_002353.2 |
| Endogenous | TAL1 | NM_003189.2 |
| Endogenous | TBX1 | NM_080646.1 |
| Endogenous | TBX4 | NM_018488.2 |
| Endogenous | TBXA2R | NM_001060.3 |
| Endogenous | TCEB1 | NM_001204857.1 |
| Endogenous | TCEB2 | NM_007108.2 |
| Endogenous | TCF20 | NM_005650.1 |
| Endogenous | TCF3 | NM_003200.3 |
| Endogenous | TCF4 | NM_003199.1 |
| Endogenous | TDGF1 | NM_003212.2 |
| Endogenous | TEK | NM_000459.3 |
| Endogenous | TF | NM_001063.2 |
| Endogenous | TFDP1 | NM_007111.4 |
| Endogenous | TFPI2 | NM_006528.3 |
| Endogenous | TGFB1 | NM_000660.3 |
| Endogenous | TGFB2 | NM_003238.2 |
| Endogenous | TGFBI | NM_000358.2 |
| Endogenous | TGFBR2 | NM_001024847.1 |
| Endogenous | THBS1 | NM_003246.2 |
| Endogenous | THBS2 | NM_003247.2 |
| Endogenous | THBS4 | NM_003248.3 |
| Endogenous | THY1 | NM_006288.2 |
| Endogenous | TIE1 | NM_005424.2 |
| Endogenous | TIMP1 | NM_003254.2 |
| Endogenous | TIMP2 | NM_003255.4 |
| Endogenous | TIMP4 | NM_003256.2 |
| Endogenous | TJP2 | NM_004817.2 |
| Endogenous | TJP3 | NM_014428.1 |
| Endogenous | TLR4 | NR_024168.1 |
| Endogenous | TMC6 | NM_001127198.1 |
| Endogenous | TMEM100 | NM_018286.2 |
| Endogenous | TMEM30B | NM_001017970.2 |
| Endogenous | TMPRSS2 | NM_005656.3 |
| Endogenous | TMPRSS4 | NM_019894.3 |
| Endogenous | TMPRSS6 | NM_153609.2 |
| Endogenous | TNC | NM_002160.3 |
| Endogenous | TNF | NM_000594.2 |
| Endogenous | TNFRSF12A | NM_016639.1 |
| Endogenous | TNFRSF1A | NM_001065.2 |
| Endogenous | TNFSF10 | NM_003810.2 |
| Endogenous | TNFSF12 | NM_003809.2 |
| Endogenous | TNFSF13 | NM_003808.3 |
| Endogenous | TNMD | NM_022144.2 |
| Endogenous | TNN | NM_022093.1 |
| Endogenous | TNS1 | NM_022648.4 |
| Endogenous | TNXB | NM_032470.3 |
| Endogenous | TOM1L1 | NM_005486.2 |
| Endogenous | TP53 | NM_000546.2 |
| Endogenous | TPM2 | NM_003289.3 |
| Endogenous | TPSB2 | NM_024164.5 |
| Endogenous | TPSD1 | NM_012217.2 |
| Endogenous | TSHR | NM_001018036.2 |
| Endogenous | TSPAN1 | NM_005727.2 |
| Endogenous | TWIST1 | NM_000474.3 |
| Endogenous | TWIST2 | NM_057179.2 |
| Endogenous | TXNIP | NM_006472.1 |
| Endogenous | TYMP | NM_001953.3 |
| Endogenous | UBA52 | NM_003333.3 |
| Endogenous | UTS2 | NM_006786.3 |
| Endogenous | VAMP8 | NM_003761.3 |
| Endogenous | VASH1 | NM_014909.4 |
| Endogenous | VAV2 | NM_003371.3 |
| Endogenous | VAV3 | NM_001079874.1 |
| Endogenous | VCAM1 | NM_001078.3 |
| Endogenous | VCAN | NM_004385.3 |
| Endogenous | VEGFA | NM_001025366.1 |
| Endogenous | VEGFB | NM_003377.3 |
| Endogenous | VEGFC | NM_005429.2 |
| Endogenous | VEZF1 | NM_007146.2 |
| Endogenous | VHL | NM_000551.2 |
| Endogenous | VIM | NM_003380.2 |
| Endogenous | VIT | NM_053276.3 |
| Endogenous | VPS13A | NM_033305.2 |
| Endogenous | VSIG4 | NM_001100431.1 |
| Endogenous | VWA1 | NM_199121.2 |
| Endogenous | VWA2 | NM_001272046.1 |
| Endogenous | WARS | NM_004184.3 |
| Endogenous | WIPF1 | NM_001077269.1 |
| Endogenous | WNT5A | NM_003392.3 |
| Endogenous | WNT5B | NM_032642.2 |
| Endogenous | WWTR1 | NM_001168278.1 |
| Endogenous | ZC3H12A | NM_025079.2 |
| Endogenous | ZCCHC24 | XM_005269604.1 |
| Endogenous | ZEB1 | NM_001128128.1 |
| Endogenous | ZEB2 | NM_014795.3 |
| Endogenous | ZFPM2 | NM_012082.3 |
| Endogenous | ZFYVE16 | NM_001105251.2 |
| Endogenous | ZFYVE9 | NM_004799.2 |
| Housekeeping | AGK | NM_018238.3 |
| Housekeeping | AMMECR1L | NM_001199140.1 |
| Housekeeping | CC2D1B | NM_032449.2 |
| Housekeeping | CNOT10 | NM_001256741.1 |
| Housekeeping | CNOT4 | NM_001190848.1 |
| Housekeeping | COG7 | NM_153603.3 |
| Housekeeping | DDX50 | NM_024045.1 |
| Housekeeping | DHX16 | NM_001164239.1 |
| Housekeeping | DNAJC14 | NM_032364.5 |
| Housekeeping | EDC3 | NM_001142443.1 |
| Housekeeping | EIF2B4 | NM_172195.3 |
| Housekeeping | ERCC3 | NM_000122.1 |
| Housekeeping | FCF1 | NM_015962.4 |
| Housekeeping | GPATCH3 | NM_022078.2 |
| Housekeeping | HDAC3 | NM_003883.2 |
| Housekeeping | MRPS5 | NM_031902.3 |
| Housekeeping | MTMR14 | NM_022485.3 |
| Housekeeping | NOL7 | NM_016167.3 |
| Housekeeping | NUBP1 | NM_001278506.1 |
| Housekeeping | PRPF38A | NM_032864.3 |
| Housekeeping | SAP130 | NM_024545.3 |
| Housekeeping | SF3A3 | NM_006802.2 |
| Housekeeping | TLK2 | NM_006852.2 |
| Housekeeping | TMUB2 | NM_024107.2 |
| Housekeeping | TRIM39 | NM_021253.3 |
| Housekeeping | USP39 | NM_001256725.1 |
| Housekeeping | ZC3H14 | NM_001160103.1 |
| Housekeeping | ZKSCAN5 | NM_014569.3 |
| Housekeeping | ZNF143 | NM_003442.5 |
| Housekeeping | ZNF346 | NM_012279.2 |
|  |  |  |
