# Supplementary material for: Circulating tumor cell and cell-free RNA capture and expression analysis identify platelet-associated genes in metastatic lung cancer
Source: BMC Cancer. 2019 Jun 19;19:603. doi: 10.1186/s12885-019-5795-x (PMC6582501; doi:10.1186/s12885-019-5795-x)
Supplement: Supplementary file 5 — Table S2. Patient and tumor characteristics. (DOCX 17 kb) [file 12885_2019_5795_MOESM5_ESM.docx]

**Table S2. Patient and tumor characteristics.**

|  | **NSCLC** | **SCLC** |
| --- | --- | --- |
| **Age** at baseline, median (range), yrs | 63 (48-76) | 62 (49-79) |
|  |  |  |
| **Gender** |  |  |
| Male | 5 (50%) | 4 (40%) |
| Female | 5 (50%) | 6 (60%) |
|  |  |  |
| **Race** |  |  |
| Caucasian | 10 (100%) | 9 (90%) |
| Other |  | 1 (10%) |
|  |  |  |
| **Histology** |  |  |
| Adenocarcinoma | 9 (90%) | 10 (100%) |
| Squamous cell carcinoma | 1 (10%) |  |
|  |  |  |
| **Number of metastatic sites** |  |  |
| 1 site | 9 (90%) | 6 (60%) |
| > 1 site | 1 (10%) | 4 (40%) |
|  |  |  |
| **Smoking** |  |  |
| None | 2 (20%) |  |
| Current/Former | 8 (80%) | 10 (100%) |
|  |  |  |
| **Prior therapy** |  |  |
| Chemonaive | 4 (40%) | 7 (70%) |
| Prior chemotherapy | 6 (60%) | 3 (30%) |
