# Supplementary material for: Circulating tumor cell and cell-free RNA capture and expression analysis identify platelet-associated genes in metastatic lung cancer
Source: BMC Cancer. 2019 Jun 19;19:603. doi: 10.1186/s12885-019-5795-x (PMC6582501; doi:10.1186/s12885-019-5795-x)
Supplement: Supplementary file 6 — Table S3. Complete list of known proteins/compounds involved in platelet degranulation. Data downloaded from Reactome. (DOCX 29 kb) [file 12885_2019_5795_MOESM6_ESM.docx]

**Table S3. Complete list of known proteins/compounds involved in platelet degranulation. Data downloaded from Reactome.**^34^

| **MoleculeType** | **Identifier** | **Molecule Name** |
| --- | --- | --- |
| Chemical Compounds | 18303 | phosphatidyl-L-serine [ChEBI:18303] |
| Chemical Compounds | 17815 | 1,2-diacyl-sn-glycerol [ChEBI:17815] |
| Chemical Compounds | 15422 | ATP [ChEBI:15422] |
| Chemical Compounds | 15996 | GTP [ChEBI:15996] |
| Chemical Compounds | 16761 | ADP [ChEBI:16761] |
| Chemical Compounds | 18420 | magnesium(2+) [ChEBI:18420] |
| Chemical Compounds | 18367 | phosphate(3-) [ChEBI:18367] |
| Chemical Compounds | 29108 | calcium(2+) [ChEBI:29108] |
| Chemical Compounds | 28790 | serotonin [ChEBI:28790] |
| Chemical Compounds | 17552 | GDP [ChEBI:17552] |
| Chemical Compounds | 29888 | diphosphoric acid [ChEBI:29888] |
| Proteins | Q12846 | UniProt:Q12846 STX4 |
| Proteins | P62937 | UniProt:P62937 PPIA |
| Proteins | Q01518 | UniProt:Q01518 CAP1 |
| Proteins | P23528 | UniProt:P23528 CFL1 |
| Proteins | P07737 | UniProt:P07737 PFN1 |
| Proteins | O43852 | UniProt:O43852 CALU |
| Proteins | P13473 | UniProt:P13473 LAMP2 |
| Proteins | P08962 | UniProt:P08962 CD63 |
| Proteins | P16109 | UniProt:P16109 SELP |
| Proteins | O00194 | UniProt:O00194 RAB27B |
| Proteins | P11021 | UniProt:P11021 HSPA5 |
| Proteins | P21333 | UniProt:P21333 FLNA |
| Proteins | Q9BYX7 | UniProt:Q9BYX7 POTEKP |
| Proteins | Q8WZ42 | UniProt:Q8WZ42 TTN |
| Proteins | P18206 | UniProt:P18206 VCL |
| Proteins | P55145 | UniProt:P55145 MANF |
| Proteins | P08758 | UniProt:P08758 ANXA5 |
| Proteins | P0DP23 | UniProt:P0DP23 CALM1 |
| Proteins | Q15833 | UniProt:Q15833 STXBP2 |
| Proteins | P68366 | UniProt:P68366 TUBA4A |
| Proteins | P00441 | UniProt:P00441 SOD1 |
| Proteins | O75083 | UniProt:O75083 WDR1 |
| Proteins | Q5JVS0 | UniProt:Q5JVS0 HABP4 |
| Proteins | O94919 | UniProt:O94919 ENDOD1 |
| Proteins | P37802 | UniProt:P37802 TAGLN2 |
| Proteins | Q9ULD4 | UniProt:Q9ULD4 BRPF3 |
| Proteins | Q9Y490 | UniProt:Q9Y490 TLN1 |
| Proteins | P08567 | UniProt:P08567 PLEK |
| Proteins | Q06481 | UniProt:Q06481 APLP2 |
| Proteins | Q6ZUX7 | UniProt:Q6ZUX7 LHFPL2 |
| Proteins | P21926 | UniProt:P21926 CD9 |
| Proteins | Q96JJ7 | UniProt:Q96JJ7 TMX3 |
| Proteins | O60245 | UniProt:O60245 PCDH7 |
| Proteins | Q6YHK3 | UniProt:Q6YHK3 CD109 |
| Proteins | P05106 | UniProt:P05106 ITGB3 |
| Proteins | P08514 | UniProt:P08514 ITGA2B |
| Proteins | O75167 | UniProt:O75167 PHACTR2 |
| Proteins | Q5SQ64 | UniProt:Q5SQ64 LY6G6F |
| Proteins | Q96C24 | UniProt:Q96C24 SYTL4 |
| Proteins | P16671 | UniProt:P16671 CD36 |
| Proteins | P16284 | UniProt:P16284 PECAM1 |
| Proteins | Q9UHQ9 | UniProt:Q9UHQ9 CYB5R1 |
| Proteins | O15439 | UniProt:O15439 ABCC4 |
| Proteins | P07602 | UniProt:P07602 PSAP |
| Proteins | Q9BWS9 | UniProt:Q9BWS9 CHID1 |
| Proteins | O00186 | UniProt:O00186 STXBP3 |
| Proteins | P17252 | UniProt:P17252 PRKCA |
| Proteins | P05771 | UniProt:P05771 PRKCB |
| Proteins | P05129 | UniProt:P05129 PRKCG |
| Proteins | P05155 | UniProt:P05155 SERPING1 |
| Proteins | O43707 | UniProt:O43707 ACTN4 |
| Proteins | P35609 | UniProt:P35609 ACTN2 |
| Proteins | P12814 | UniProt:P12814 ACTN1 |
| Proteins | P04196 | UniProt:P04196 HRG |
| **Proteins** | **P09486** | **UniProt:P09486 SPARC** |
| Proteins | P00746 | UniProt:P00746 CFD |
| Proteins | P05121 | UniProt:P05121 SERPINE1 |
| Proteins | P02775 | UniProt:P02775 PPBP |
| Proteins | Q13201 | UniProt:Q13201 MMRN1 |
| Proteins | P00451 | UniProt:P00451 F8 |
| Proteins | P04275 | UniProt:P04275 VWF |
| **Proteins** | **P02776** | **UniProt:P02776 PF4** |
| Proteins | P02751 | UniProt:P02751 FN1 |
| Proteins | P08697 | UniProt:P08697 SERPINF2 |
| Proteins | P01133 | UniProt:P01133 EGF |
| Proteins | P14210 | UniProt:P14210 HGF |
| Proteins | P01009 | UniProt:P01009 SERPINA1 |
| Proteins | P04217 | UniProt:P04217 A1BG |
| Proteins | P62328 | UniProt:P62328 TMSB4X |
| Proteins | P02671 | UniProt:P02671 FGA |
| Proteins | P02679 | UniProt:P02679 FGG |
| Proteins | P02675 | UniProt:P02675 FGB |
| Proteins | P00488 | UniProt:P00488 F13A1 |
| Proteins | P07996 | UniProt:P07996 THBS1 |
| Proteins | Q14393 | UniProt:Q14393 GAS6 |
| Proteins | P05067 | UniProt:P05067 APP |
| **Proteins** | **P10124** | **UniProt:P10124 SRGN** |
| Proteins | O00391 | UniProt:O00391 QSOX1 |
| Proteins | P02765 | UniProt:P02765 AHSG |
| Proteins | P02768 | UniProt:P02768 ALB |
| Proteins | P01042 | UniProt:P01042 KNG1 |
| Proteins | P01033 | UniProt:P01033 TIMP1 |
| Proteins | P04075 | UniProt:P04075 ALDOA |
| **Proteins** | **P10909** | **UniProt:P10909 CLU** |
| Proteins | P19652 | UniProt:P19652 ORM2 |
| Proteins | P02763 | UniProt:P02763 ORM1 |
| Proteins | Q9BX10 | UniProt:Q9BX10 GTPBP2 |
| Proteins | Q9Y6I9 | UniProt:Q9Y6I9 TEX264 |
| Proteins | Q9NTK5 | UniProt:Q9NTK5 OLA1 |
| Proteins | P12259 | UniProt:P12259 F5 |
| Proteins | Q9NXH8 | UniProt:Q9NXH8 TOR4A |
| Proteins | P07225 | UniProt:P07225 PROS1 |
| Proteins | O00292 | UniProt:O00292 LEFTY2 |
| Proteins | Q8NBX0 | UniProt:Q8NBX0 SCCPDH |
| Proteins | O14498 | UniProt:O14498 ISLR |
| Proteins | Q86UX7 | UniProt:Q86UX7 FERMT3 |
| Proteins | P00747 | UniProt:P00747 PLG |
| Proteins | P01023 | UniProt:P01023 A2M |
| Proteins | P04085-1 | UniProt:P04085-1 PDGFA |
| Proteins | P01127 | UniProt:P01127 PDGFB |
| Proteins | Q8NBF2 | UniProt:Q8NBF2 NHLRC2 |
| Proteins | Q6UXV4 | UniProt:Q6UXV4 APOOL |
| Proteins | Q9UEU0 | UniProt:Q9UEU0 VTI1B |
| Proteins | P05019 | UniProt:P05019 IGF1 |
| Proteins | P01344 | UniProt:P01344 IGF2 |
| Proteins | P10600 | UniProt:P10600 TGFB3 |
| Proteins | P61812 | UniProt:P61812 TGFB2 |
| Proteins | P01137 | UniProt:P01137 TGFB1 |
| Proteins | Q9UNF1 | UniProt:Q9UNF1 MAGED2 |
| Proteins | Q9NUQ9 | UniProt:Q9NUQ9 FAM49B |
| Proteins | O43915 | UniProt:O43915 VEGFD |
| Proteins | P49765 | UniProt:P49765 VEGFB |
| Proteins | P15692 | UniProt:P15692 VEGFA |
| Proteins | P49767 | UniProt:P49767 VEGFC |
| Proteins | Q8NBM8 | UniProt:Q8NBM8 PCYOX1L |
| Proteins | P01011 | UniProt:P01011 SERPINA3 |
| Proteins | Q7L3B6 | UniProt:Q7L3B6 CDC37L1 |
| Proteins | P02749 | UniProt:P02749 APOH |
| Proteins | Q13103 | UniProt:Q13103 SPP2 |
| Proteins | P49908 | UniProt:P49908 SELENOP |
| Proteins | P29622 | UniProt:P29622 SERPINA4 |
| Proteins | Q06033 | UniProt:Q06033 ITIH3 |
| Proteins | P35625 | UniProt:P35625 TIMP3 |
| Proteins | Q08380 | UniProt:Q08380 LGALS3BP |
| Proteins | Q92520 | UniProt:Q92520 FAM3C |
| Proteins | Q99969 | UniProt:Q99969 RARRES2 |
| Proteins | Q16610 | UniProt:Q16610 ECM1 |
| Proteins | Q14624 | UniProt:Q14624 ITIH4 |
| Proteins | P56202 | UniProt:P56202 CTSW |
| Proteins | P05452 | UniProt:P05452 CLEC3B |
| Proteins | P02647 | UniProt:P02647 APOA1 |
| Proteins | Q8WXD2 | UniProt:Q8WXD2 SCG3 |
| Proteins | P02787 | UniProt:P02787 TF |
