# Supplementary material for: Circulating tumor cell and cell-free RNA capture and expression analysis identify platelet-associated genes in metastatic lung cancer
Source: BMC Cancer. 2019 Jun 19;19:603. doi: 10.1186/s12885-019-5795-x (PMC6582501; doi:10.1186/s12885-019-5795-x)
Supplement: Supplementary file 7 — Table S4. List of known proteins associated with platelet alpha granule content release. Data downloaded from Reactome. (DOCX 22 kb) [file 12885_2019_5795_MOESM7_ESM.docx]

**Table S4. List of known proteins associated with platelet alpha granule content release. Data downloaded from Reactome.**^31^

| **Molecule type** | **Identifier** | **Molecule name** |
| --- | --- | --- |
| Proteins | P05155 | UniProt:P05155 SERPING1 |
| Proteins | O43707 | UniProt:O43707 ACTN4 |
| Proteins | P35609 | UniProt:P35609 ACTN2 |
| Proteins | P12814 | UniProt:P12814 ACTN1 |
| Proteins | P04196 | UniProt:P04196 HRG |
| **Proteins** | **P09486** | **UniProt:P09486 SPARC** |
| Proteins | P00746 | UniProt:P00746 CFD |
| Proteins | P05121 | UniProt:P05121 SERPINE1 |
| Proteins | P02775 | UniProt:P02775 PPBP |
| Proteins | Q13201 | UniProt:Q13201 MMRN1 |
| Proteins | P00451 | UniProt:P00451 F8 |
| Proteins | P04275 | UniProt:P04275 VWF |
| **Proteins** | **P02776** | **UniProt:P02776 PF4** |
| Proteins | P02751 | UniProt:P02751 FN1 |
| Proteins | P08697 | UniProt:P08697 SERPINF2 |
| Proteins | P01133 | UniProt:P01133 EGF |
| Proteins | P14210 | UniProt:P14210 HGF |
| Proteins | P01009 | UniProt:P01009 SERPINA1 |
| Proteins | P04217 | UniProt:P04217 A1BG |
| Proteins | P62328 | UniProt:P62328 TMSB4X |
| Proteins | P02671 | UniProt:P02671 FGA |
| Proteins | P02679 | UniProt:P02679 FGG |
| Proteins | P02675 | UniProt:P02675 FGB |
| Proteins | P00488 | UniProt:P00488 F13A1 |
| Proteins | P07996 | UniProt:P07996 THBS1 |
| Proteins | Q14393 | UniProt:Q14393 GAS6 |
| Proteins | P05067 | UniProt:P05067 APP |
| **Proteins** | **P10124** | **UniProt:P10124 SRGN** |
| Proteins | O00391 | UniProt:O00391 QSOX1 |
| Proteins | P02765 | UniProt:P02765 AHSG |
| Proteins | P02768 | UniProt:P02768 ALB |
| Proteins | P01042 | UniProt:P01042 KNG1 |
| Proteins | P01033 | UniProt:P01033 TIMP1 |
| Proteins | P04075 | UniProt:P04075 ALDOA |
| **Proteins** | **P10909** | **UniProt:P10909 CLU** |
| Proteins | P19652 | UniProt:P19652 ORM2 |
| Proteins | P02763 | UniProt:P02763 ORM1 |
| Proteins | Q9BX10 | UniProt:Q9BX10 GTPBP2 |
| Proteins | Q9Y6I9 | UniProt:Q9Y6I9 TEX264 |
| Proteins | Q9NTK5 | UniProt:Q9NTK5 OLA1 |
| Proteins | P12259 | UniProt:P12259 F5 |
| Proteins | Q9NXH8 | UniProt:Q9NXH8 TOR4A |
| Proteins | P07225 | UniProt:P07225 PROS1 |
| Proteins | O00292 | UniProt:O00292 LEFTY2 |
| Proteins | Q8NBX0 | UniProt:Q8NBX0 SCCPDH |
| Proteins | O14498 | UniProt:O14498 ISLR |
| Proteins | Q86UX7 | UniProt:Q86UX7 FERMT3 |
| Proteins | P00747 | UniProt:P00747 PLG |
| Proteins | P01023 | UniProt:P01023 A2M |
| Proteins | P04085-1 | UniProt:P04085-1 PDGFA |
| Proteins | P01127 | UniProt:P01127 PDGFB |
| Proteins | Q8NBF2 | UniProt:Q8NBF2 NHLRC2 |
| Proteins | Q6UXV4 | UniProt:Q6UXV4 APOOL |
| Proteins | Q9UEU0 | UniProt:Q9UEU0 VTI1B |
| Proteins | P05019 | UniProt:P05019 IGF1 |
| Proteins | P01344 | UniProt:P01344 IGF2 |
| Proteins | P10600 | UniProt:P10600 TGFB3 |
| Proteins | P61812 | UniProt:P61812 TGFB2 |
| Proteins | P01137 | UniProt:P01137 TGFB1 |
| Proteins | Q9UNF1 | UniProt:Q9UNF1 MAGED2 |
| Proteins | Q9NUQ9 | UniProt:Q9NUQ9 FAM49B |
| Proteins | O43915 | UniProt:O43915 VEGFD |
| Proteins | P49765 | UniProt:P49765 VEGFB |
| Proteins | P15692 | UniProt:P15692 VEGFA |
| Proteins | P49767 | UniProt:P49767 VEGFC |
| Proteins | Q8NBM8 | UniProt:Q8NBM8 PCYOX1L |
| Proteins | P01011 | UniProt:P01011 SERPINA3 |
